# A Multifunctional Anti-Proton Electrolyte for High-Rate and Super-Stable Aqueous Zn-Vanadium Oxide Battery

**DOI:** 10.1007/s40820-022-00907-4

**Published:** 2022-08-02

**Authors:** Yangwu Chen, Dingtao Ma, Kefeng Ouyang, Ming Yang, Sicheng Shen, Yanyi Wang, Hongwei Mi, Lingna Sun, Chuanxin He, Peixin Zhang

**Affiliations:** 1 College of Chemistry and Environmental Engineering, Shenzhen University, Shenzhen, 518060 People’s Republic of China; 2 Institute of Microscale Optoelectronics, Shenzhen University, Shenzhen, 518060 People’s Republic of China

**Keywords:** Zn-vanadium oxide battery, Multifunctional anti-proton electrolyte, Integrated synergetic modification, “All-in-one” solution

## Abstract

Large volumetric expansion of cathode hosts and sluggish transport kinetics in the cathode–electrolyte interface, as well as dendrite growth and hydrogen evolution at Zn anode side are considered as the system problems that cause the electrochemical failure of aqueous Zn-vanadium oxide battery. In this work, a multifunctional anti-proton electrolyte was proposed to synchronously solve all those issues. Theoretical and experimental studies confirm that PEG 400 additive can regulate the Zn^2+^ solvation structure and inhibit the ionization of free water molecules of the electrolyte. Then, smaller lattice expansion of vanadium oxide hosts and less associated by-product formation can be realized by using such electrolyte. Besides, such electrolyte is also beneficial to guide the uniform Zn deposition and suppress the side reaction of hydrogen evolution. Owing to the integrated synergetic modification, a high-rate and ultrastable aqueous Zn-V_2_O_3_/C battery can be constructed, which can remain a specific capacity of 222.8 mAh g^−1^ after 6000 cycles at 5 A g^−1^, and 121.8 mAh g^−1^ even after 18,000 cycles at 20 A g^−1^, respectively. Such “all-in-one” solution based on the electrolyte design provides a new strategy for developing high-performance aqueous Zn-ion battery.

**Supplementary Information:**

The online version contains supplementary material available at 10.1007/s40820-022-00907-4.

## Highlights

- The introduction of PEG 400 additive in the aqueous electrolyte enables regulating the Zn^2+^ solvation structure and inhibiting the ionization of free water molecules.
- Such anti-proton electrolyte can not only reduce the lattice expansion of cathode hosts and inhibit the associated by-products, but also guide the uniform Zn deposition and inhibit the hydrogen evolution reaction.
- A high-rate Zn-V_2_O_3_/C battery with 18,000-cycle shelf-life can be demonstrated via the integrated synergetic modification mechanism.

**Supplementary Information:**

The online version contains supplementary material available at 10.1007/s40820-022-00907-4.

## Graphical abstract

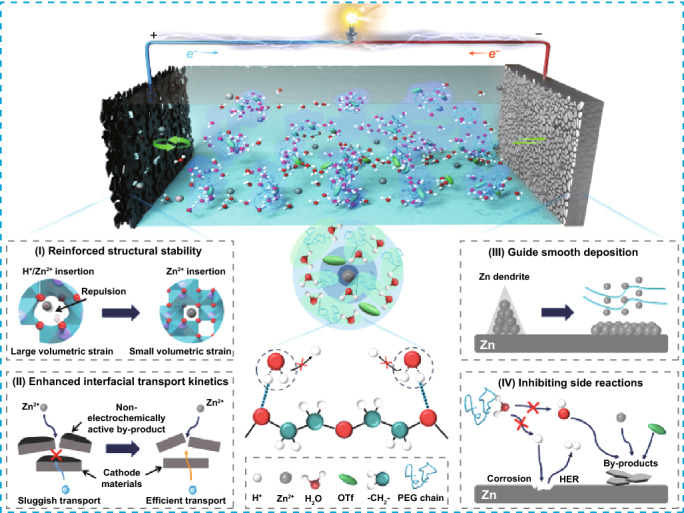

## Introduction

The increasing energy crisis and environmental pollution promote the continuous exploration of green energy storage solution. Rechargeable aqueous zinc-ion batteries (AZIBs) are supposed to be the potential next-generation candidate benefited from the low cost, intrinsic safety and high theoretical capacity, which have received extensive attention in the recent years [1–3]. To develop high-rate and long shelf-life AZIBs, many kinds of cathode materials have been successively demonstrated, which mainly includes the manganese-based oxides [4–7], vanadium-based compounds [8, 9], Prussian blue analogues (PBAs) [10, 11] and organic materials [12]. Among them, vanadium oxides (such as V_2_O_5_, VO_2_, V_2_O_3_) are considered as the promising storage host due to the abundant valence state to achieve high specific capacity, and open-frameworks assembled by various coordination polyhedral to facilitate the efficient ion storage during the electrochemical cycling [13, 14]. However, it has been found that such aqueous zinc-vanadium oxide battery in the acidic electrolyte usually suffers from insufficient rate performance and poor cycle lifespan (< 3000 cycles) in the research, which greatly hinders their practical applications [15, 16].

**Scheme 1:**
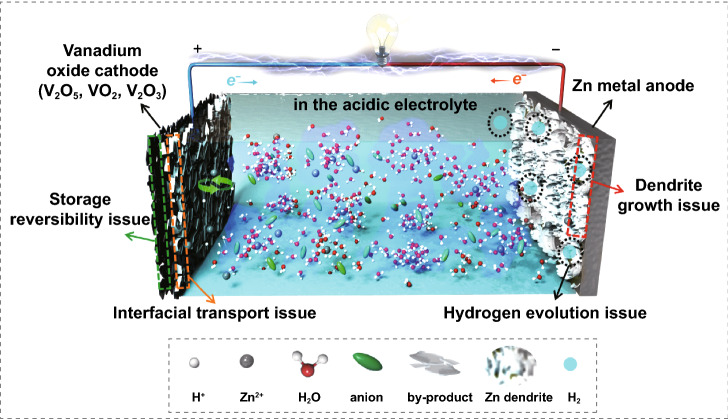
Illustration of the electrochemical failure model of aqueous zinc-vanadium oxide battery

Generally, it should be a system problem that causes the electrochemical failure of aqueous zinc-vanadium oxide battery, as shown in Scheme 1, the adverse factors can be mainly divided into the following two concerns. For Zn metal anode side, on one hand, the uneven deposition would lead to the rapid growth of Zn dendrite and produce the risk of puncturing the glass fiber (GF) separator [17, 18]. Besides that, the side reactions especially for hydrogen evolution reaction (HER) would lead to a sharp increment of internal pressure in battery [19–21]. While for the vanadium oxide cathode side, from the perspective of storage mechanism, the co-embedding of Zn^2+^/H^+^ into vanadium oxide host and accompanying with the generation of alkali by-product in the cathode/electrolyte interface is the most commonly acknowledged storage mechanism especially in the acidic electrolyte (Zn(OTf)_2_/H_2_O) system [22, 23]. Note that such low conductive by-product would not only consume the zinc ions in the electrolyte, but also block the interfacial ion/electron transport during cycling [24]. In this case, sluggish interfacial transport and low storage reversibility should be the most critical issues. Therefore, how to simultaneously solve all these faced issues is the key point to develop high-performance zinc-vanadium oxide battery yet still to be a research challenge.

Reviewing the research progress, most of the previous reports only focus on either the cathode side or anode side, while less attention has been paid for the integrated synergetic modification of both of them. For example, in order to improve the storage reversibility and interfacial transport kinetics of vanadium oxide cathode materials, a series of modification strategies such as surface coating [25], composite formation [26], ions intercalation [27], and morphological design [28] have been previously proposed. However, those approaches usually require the complex process; moreover, it is also being difficult for those simplex modifications pathway to concurrently ensure the rapid interfacial transport kinetics and highly reversible ions insertion/extraction for host framework. This is because the repulsion of H^+^ and Zn^2+^ during insertion process would induce the large lattice expansion and the accumulation of volumetric strain after long-term cycling would damage the host framework. Meanwhile, the residual by-product covered the surface of active materials would still impede the ion transport and electron transfer and finally weaken the storage performance of battery. Considering the adverse chain effect caused by proton participation during storage reactions, the suppress of proton electrochemistry should be the fundamental way to solve the above questions. To the best of our knowledge, only few published reports were focused on this research area and more deep works are still desirable. Besides, the exploration of brand-new “all-in-one” solution as universal way to enabling high-performance aqueous zinc-ion battery is also highly eager.

Herein, for the first time, we report the strategy of multifunctional anti-proton electrolyte to enabling high-rate and ultralong shelf-life aqueous zinc-vanadium oxide battery. Experimental and molecular dynamics studies confirmed that the electrochemical activity of proton in the modified electrolyte can be effectively inhibited through trap and isolate the free water molecules by the PEG molecular chain. On this basis, *in-situ* XRD and *in-situ* EIS research further reveals that the proton insertion behavior and the formation of by-product can be maximally inhibited in such anti-proton electrolyte, thus significant decreasing the lattice expansion of host framework and facilitating the interfacial transport kinetics. Moreover, such electrolyte is also beneficial to inhibit the H_2_ evolution and guide the smooth Zn deposition. As a result, benefited from the integrated synergetic modification mechanism enabled by such multifunctional anti-proton electrolyte, the assembled aqueous Zn-V_2_O_3_/C battery performs a significantly enhanced rate performance, and the cyclic stability can be even extended up to 18,000 cycles with the coulomb efficiency of nearly 100% at the ultrahigh current density of 20 A g^−1^.

## Experimental Section

### Materials Preparation

V_2_AlC (11 technology co.,LTD.), 30% H_2_O_2_ (XILONG SCIENTIFIC), Zn(OTf)_2_ (> 98%, TCI), LiF (99.9% Macklin), 12 mol mL^−1^ HCL (38 wt% Hushi reagent), H_2_C_2_O_4_ (> 99.5%, Tianjin Baishi Chemical), V_2_O_5_ (99%, Xiya reagent), polyethylene glycol 400 (PEG 400, TCI), polyvinylidene fluoride (PVDF, arkema), acetylene black (KJ MTI), and 1-Methyl-2-pyrrolidinone (NMP, > 98%, Aladdin) were purchased and directly used without further purification.

### Materials Synthesis

#### ***Synthesis of V***_***2***_***CT***_***x***_*** MXene Nanosheets***

V_2_CT_x_ nanosheets were prepared via etching the V_2_AlC powder by using lithium fluoride (LiF)/hydrochloric acid (HCl) solution. In detail, 1.0 g of V_2_AlC powder was slowly added into LiF/HCl solution (2.0 g LiF dispersed into 30 mL of 12 M HCl) within 10 min, which was continuously stirred for 0.5 h at 25 °C. Afterward, the turbid suspension was transferred to a 50 mL teflon-lined stainless-steel autoclave and sealed at 120 °C for 36 h. The turbid suspension was centrifuged to collect the sediments, followed by washing with deionized water for several times until the pH turned neutral. The sediments were further rinsed by ethanol for three times before vacuum-drying at 60 °C for 12 h, and finally receiving the V_2_CT_x_ nanosheets.

#### ***Synthesis of V***_***2***_***O***_***3***_***/C Nanosheets***

2 mL of 30 wt% H_2_O_2_ diluted into 20 mL of 3 wt% H_2_O_2_ was dropwise added into 100 mL of ~ 2.0 mg mL^−1^ V_2_CT_x_ suspension under vigorous stirring. The mixture was kept stirring for 10 min, and subsequently immersed into liquid nitrogen. After the dispersion was completely frozen, it was subjected to a vacuum freeze drier for at least 36 h. The obtained powder was annealed at 800 °C for 2 h with a ramping rate of 5 °C min^−1^ under Ar/H_2_ (95/5, vol/vol) flow, resulting in the V_2_O_3_/C nanosheets.

#### ***Synthesis of VO***_***2***_

V_2_O_5_ (1.2 g) and H_2_C_2_O_4_⋅2H_2_O (2.5 g) were initially added into deionized water (40 mL), and then the above mixture was reacted at 75 °C under magnetic stirring for 60 min to obtain a dark blue dispersion. Then, the above dispersion was transferred into a 50 mL teflon-lined autoclave and kept at 180 °C for 180 min. Finally, the product was collected and washed with ethanol and deionized water.

#### Preparation of Anti-Proton Electrolyte

A certain amount of Zn(OTf)_2_ (> 98%, TCI) was dissolved in deionized water to prepare 3 M Zn(OTf)_2_ electrolyte (denoted as 0PEG electrolyte). Correspondingly, a certain amount of Zn(OTf)_2_ was dissolved in the co-solvent of 50 wt% PEG 400 and 50 wt% deionized water to obtain the anti-proton electrolyte (denoted as 50PEG electrolyte).

### Materials Characterization

Surface morphology and energy-dispersive spectroscopy (EDS) elemental mapping were examined by field emission scanning electron microscopy (FESEM, JEOL, JSM-7800F) equipped with an energy-dispersive X-ray spectrometer (EDS) (Ametek,TEAM Octane Plus) at an accelerating voltage of 15 kV. Transmission electron microscopy (TEM) was performed using a JEM-2100 & X-Max80 microscope under an accelerating voltage of 200 kV. X-ray diffraction (XRD) measurement was performed using a PANalytical, Empyrean, CuKa radiation (λ = 1.54065 Å) Generator at 45 kV and 40 mA. Atomic force microscopy (AFM) was carried out using a Bruker dimension icon microscope with ScanAsyst. Raman spectra were recorded using Thermo Fisher Renishaw inVia spectrometer with excitation wavelength λ = 532 nm. XPS measurement was performed using the Thermo Fisher Scientific K-Alpha spectrometer (Al Kα radiation) with a scanning rate of 0.05 eV per step. Operando optical observation was carried out on Motic BA310Met coupling with a CHI760e electrochemical workstation. For the calculation of lattice expansion ratio, this value (Δ*d*/*d*) can be calculated using the parameter of the initial state (*d*) as a reference.

### Electrochemical Measurements

To prepare the cathode, active materials (V_2_O_3_/C, VO_2_ and commercial V_2_O_5_), acetylene black, and PVDF with a mass ratio of 7:2:1 were mixed in NMP under stirring. Then, the slurry was uniformly casted on the Ti foil, and immediately vacuum-drying at 60 ℃ for 12 h to thoroughly remove NMP. The as-fabricated cathode was further punched out into circular disks with a diameter of 12 mm to match the zinc foil anode, 3 M Zn(OTf)_2_/H_2_O and 3 M Zn(OTf)_2_ in H_2_O/PEG 400 with the mass ratio of 1:1 were employed as electrolyte, glass microfiber filter (Whatman GF/D) was punched out into circular disks with a diameter of 17 mm as separator.

CV test was performed at various scan rate from 0.1 to 1 mV s^−1^. Electrochemical impedance spectroscopy (EIS) was tested using 1470E electrochemical workstation (Solartron Analytical, Ametek). Galvanostatic intermittent titration technique (GITT) measurement was also executed on CT3001A cell testing system, before the GITT measurement, the assembled cells were first charged and discharged at 0.2 A g^−1^ for three cycle to stabilize the cells. The current pulse lasted for 5 min at 0.2 A g^−1^, and then the cell was relaxed for 30 min to make the voltage reach the equilibrium. The D_Zn_^2+^ value was calculated by formula (1):

1 $${\mathrm{D}}_{\mathrm{Zn}}^{ 2+}=\frac{4}{\mathrm{\pi \tau }}{\left(\frac{{\mathrm{m}}_{\mathrm{B}}{\mathrm{V}}_{\mathrm{m}}}{{\mathrm{M}}_{\mathrm{B}}\mathrm{S}}\right)}^{2}{\left(\frac{\Delta {\mathrm{E}}_{\mathrm{s}}}{\Delta {\mathrm{E}}_{\uptau }}\right)}^{2}$$

where τ was the constant current pulse time; m_B_, *M*_B_ and *V*_m_ represent the mass, molar mass and molar volume of cathode material, respectively. *S* was the effective area of the working electrode. Δ*E*τ and Δ*E*s represent the difference between the current flux and the steady-state voltage as the voltage changes during the constant current pulse, respectively.

### Molecular Dynamic Simulation

Atomistic molecular dynamics simulations have been performed in the GROMACS (version 2020.6) simulation package, using the general amber force field (gaff2) and the TIP3P water model. The amorphous polymer was built in the materials studio software and 150 Zn ions and 300 OTf^−^ molecules were randomly placed in a cubic box of around 5 nm with or without the polymer matrix. After solvation with 2778 water molecules, the systems were equilibrated through thousands of steps of energy minimization and 20 ns equilibration before the production runs of another 20 ns under the NPT ensemble. The temperature was coupled to 298 K using the Nose–Hoover method and the pressure was coupled to 1 atm using the Parrinello–Rahman method. The cutoff scheme of 1.2 nm was implemented for the non-bonded interactions, and the Particle Mesh Ewald method with a Fourier spacing of 0.1 nm was applied for the long-range electrostatic interactions. All covalent bonds with hydrogen atoms were constraint using the LINCS algorithm.

## Results and Discussion

### Solvation Structure Reorganization of Anti-Proton Electrolyte

**Fig. 1:**
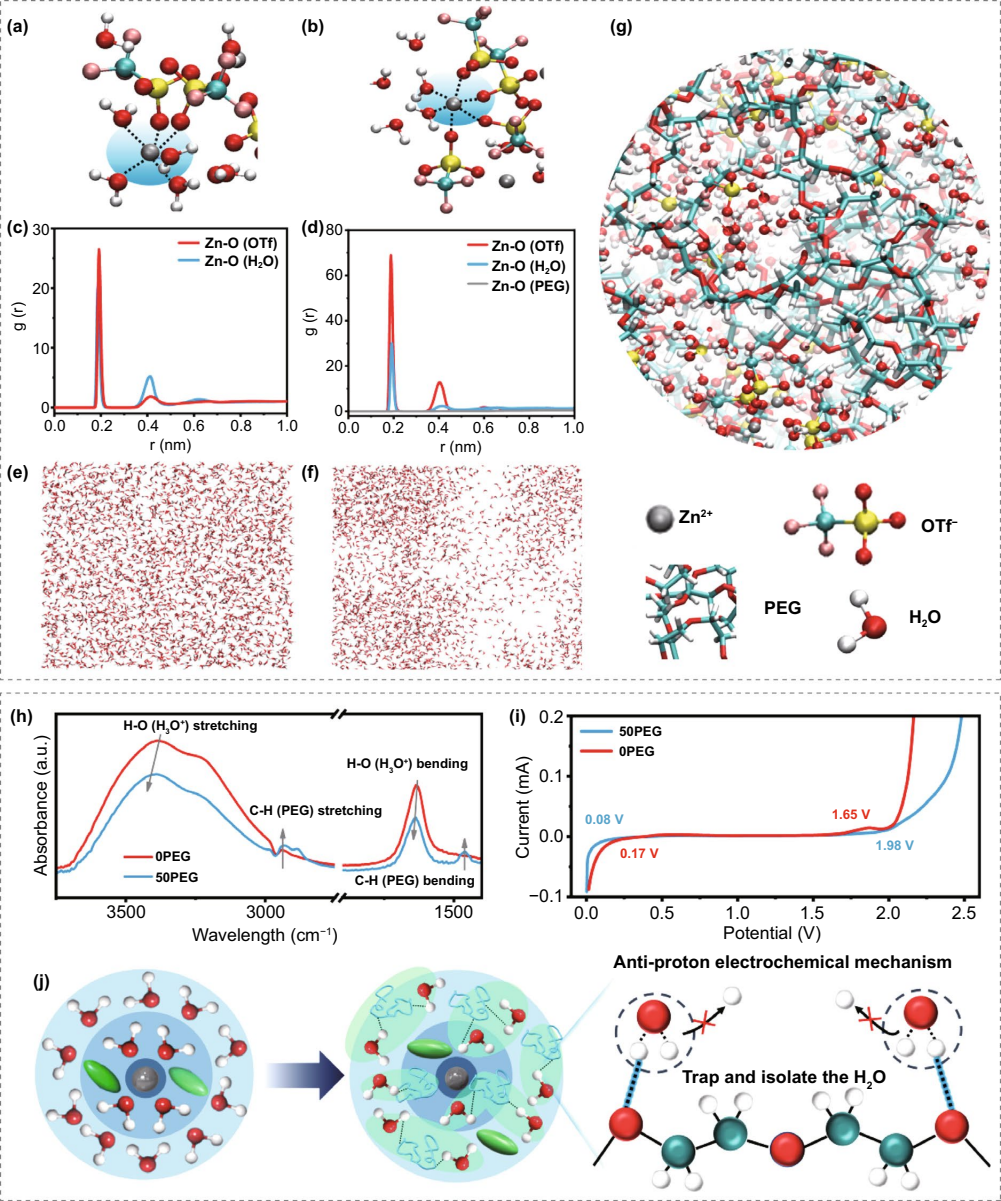
Typical Zn^2+^ solvation structure of **a** 0PEG and **b** 50PEG electrolyte. The radial distribution functions RDFs g(r) for Zn^2+^-O(H_2_O), Zn^2+^-O(OTf ^−^) and Zn^2+^-O(PEG) of the **c** 0PEG and **d** 50PEG electrolyte. Simulation snapshots of only water molecules of the **e** 0PEG and **f** 50PEG electrolyte. **g** The snapshot of water molecules absorbed by the PEG-rich regions in the 50 PEG electrolyte. **h** Normalized FTIR spectra of the 0PEG and 50PEG electrolyte. **i** LSV curves of the 0PEG and 50PEG electrolyte at a scan rate of 0.1 mV s^−1^_._ **j** The illustration of anti-proton electrochemical mechanism regulated by PEG additive modification

To explore the influence of PEG 400 additive (Fig. S1) on tuning the Zn^2+^ solvation structure as well as the electrochemical activity of free water molecules in the electrolyte, molecular dynamics (MD) simulation was carried out. As can be seen, the solvation structure of Zn^2+^ is mainly coordinated to 2 OTf^**−**^ and 4 H_2_O in 0PEG electrolyte (Fig. 1a), whereas the Zn^2+^ solvation shell typically includes 4 OTf ^−^ and 2 H_2_O in 50PEG electrolyte (Fig. 1b). Besides that, as confirmed from the radial distribution functions in Fig. 1b–d, the coordination distance of H_2_O and Zn^2+^ is shorter than OTf ^–^ in the 0PEG electrolyte, and the coordination distance between PEG and Zn^2+^ is extremely short in 50PEG electrolyte, manifesting the PEG molecular chain has the strongest interaction with the Zn^2+^ ions compared to OTf^−^ and H_2_O. Then, the snapshot of the content of free water molecules and the structure in 0PEG and 50PEG electrolyte are shown in Fig. 1e–f. It is obvious that the content of free water molecules in the 50PEG electrolyte is significantly less than that in the 0PEG electrolyte. Moreover, the snapshot of 50PEG electrolyte (Fig. 1g) reveals that the PEG molecular chain would aggregate to form the PEG-rich regions. Note that such PEG-rich regions exhibit strong adsorption capability to the free water molecules rather than Zn^2+^, which implies that the PEG molecular chains would change the hydrogen-bond structure among free water molecules owing to the strong interaction with free water molecules, thereby greatly reducing the electrochemical activity of the free water molecules in the electrolyte.

Subsequently, Fourier-transform infrared spectroscopy (FTIR) was further performed to analysis the structural change of water molecules in the electrolyte after PEG 400 additive modification (Fig. 1h). Compared with the 0PEG system, both H–O bending and H–O stretching vibration modes of water molecules would shift to higher wavenumber and accompany with the increasing strength of C-H bending and C-H stretching vibration in the 50PEG system [29, 30]. Such phenomenon should be attributed to the perturbation of water hydrogen-bond network by the as-formed H_2_O-PEG hydrogen-bond interaction [31, 32]. A lower content of ionized H^+^ in 50PEG electrolyte also can be revealed by the pH test (Fig. S2). In addition, the electrochemical stability window of 0PEG and 50PEG electrolyte was determined by linear sweep voltammetry (LSV), as shown in Fig. 1i. The result shows that the 50PEG electrolyte has a wider stable window (0.08–1.98 V) and lower corrosion current density than that of the 0PEG electrolyte (0.17–1.65 V), implying it a stronger tolerance for HER. Throughout the above research, in 50PEG electrolyte system, excepting the regulation of the solvation structure of Zn^2+^, the free water molecules would be trapped and isolated by the PEG molecular chain through forming the new hydrogen bond, especially in the PEG-rich regions, thus suppressing the ionization of free water molecules (Fig. 1j).

### Characterizations of the V_2_O_3_/C Nanosheets Cathode

**Fig. 2:**
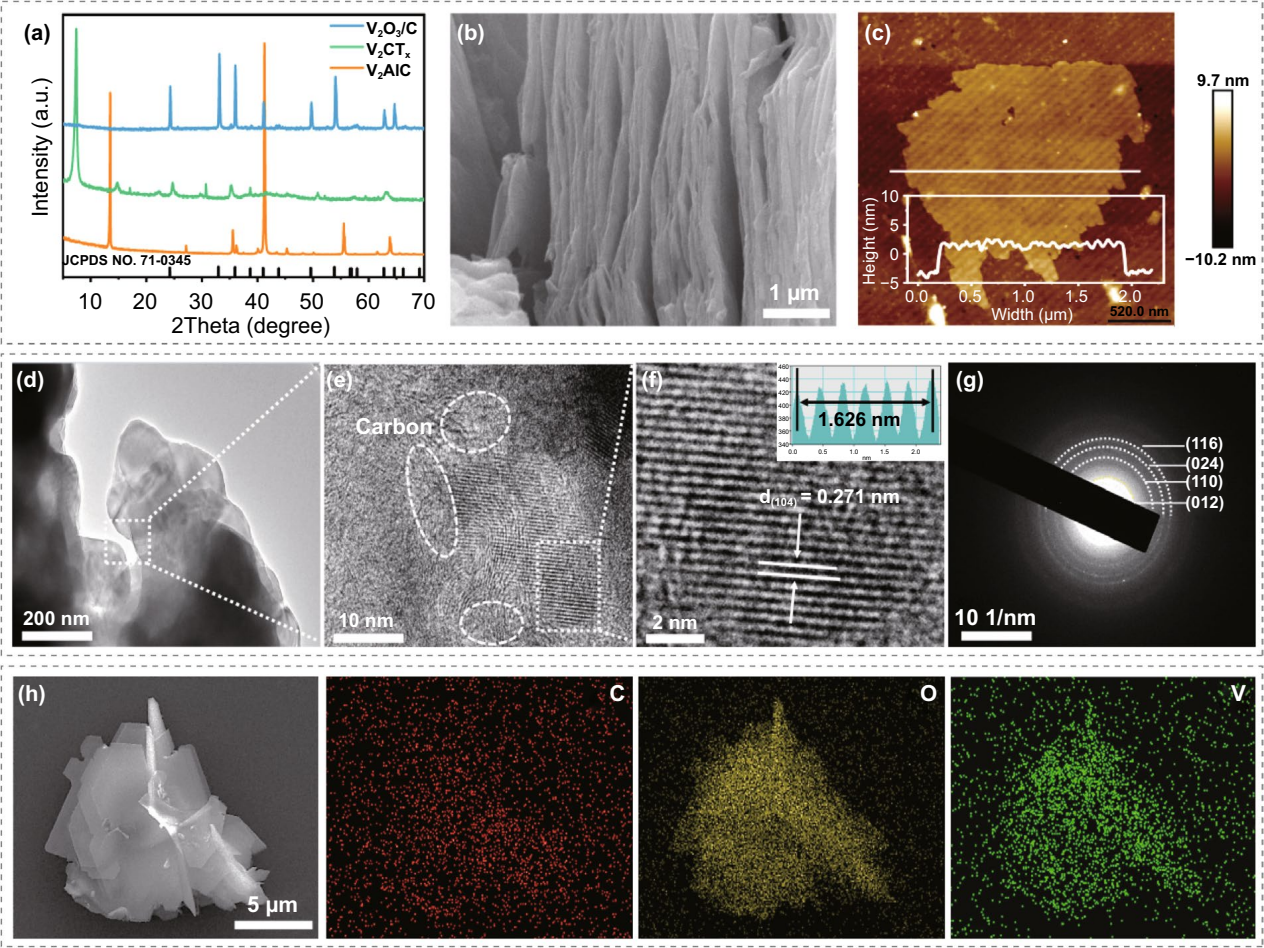
**a** XRD pattern of V_2_AlC, V_2_CT_x_ MXene and V_2_O_3_/C nanosheets. **b** FESEM image of V_2_CT_x_ MXene. **c** AFM image of the V_2_O_3_/C nanosheets. **d** TEM image and **e**, **f** corresponding HRTEM images and **g** SAED image of the V_2_O_3_/C nanosheets. **h** EDS elemental mapping of the V_2_O_3_/C nanosheets

V_2_CT_x_ MXene-derived V_2_O_3_/C nanosheets were fabricated via pre-oxidation and subsequent reduction calcination. X-ray diffraction pattern (Fig. 2a) confirms the well match of as-synthesized V_2_O_3_/C with rhombohedral V_2_O_3_ phase (JPCDS NO. 71–0345) and no any other impurity peak is detected [33]. Note that such V_2_O_3_/C composite enables inheriting the morphology characteristics of V_2_CT_x_ MXene sheet (Fig. 2b) and possessing an ultrathin thickness of nearly 5 nm (Fig. 2c). TEM image (Fig. 2d) and corresponding HRTEM images (Fig. 2e–f) and selected-area electron diffraction (SAED) pattern (Fig. 2g) further demonstrate the formation of composite of amorphous carbon and high crystallinity V_2_O_3_. EDS elemental mapping (Fig. 2h) also confirms the homogeneous distribution of C, O, and V elements of V_2_O_3_/C nanosheets. Moreover, the fitted result of V 2*p* XPS peak (Fig. S3) indicates the dominant valence state of + 3 in V_2_O_3_/C with the V^3+^/V^2+^ ratio of 1.9. On the other hand, Raman spectrum (Fig. S4) of V_2_O_3_/C composite manifests the main existence of five kinds of vibration modes. Among them, the low-frequency signal peaks located at 142 and 265 cm^−1^ could be, respectively, interpreted as bending vibration of vanadium-based bonds in [VO_6_] octahedra and V = O bonds, while the signal peaks at 501 and 686 cm^−1^ should be attributed to the stretching vibration of the V–O bond, and the peak located at 1000 cm^−1^ corresponds to the stretching mode of V = O bond [34, 35].

### Electrochemical Performance of Aqueous Zn-V_2_O_3_/C Battery

**Fig. 3:**
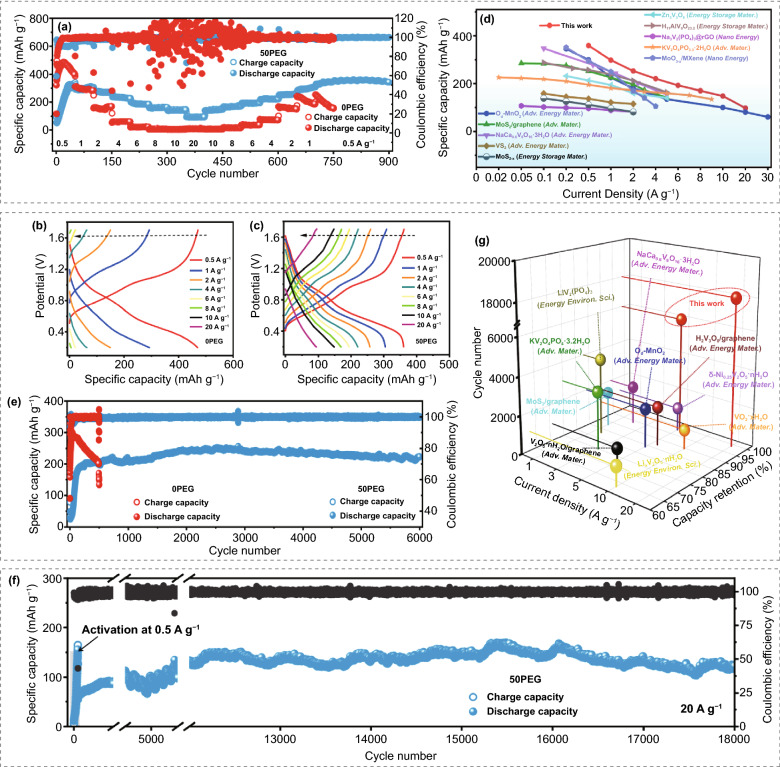
Electrochemical performance of the V_2_O_3_/C cathode. **a** Rate performance test and (**b**, **c**) corresponding charge–discharge curves at different current densities. **d** The comparison of rate performance between this work and other previous works. Cyclic stability test at the current density of **e** 5 A g^−1^ and **f** 20 A g^−1^, respectively. **g** The comparison of cyclic stability between this work and other previous reports

To reveal the influence of such anti-proton electrolyte on storage capability of V_2_O_3_/C cathode, Zn-V_2_O_3_/C battery was assembled to test the electrochemical performance. First, the rate performance test was depicted in Fig. 3a, which indicates the significantly enhanced rate capability after the electrolyte modification. In detail, compared with the low specific capacity achieved in the 0PEG system, the electrode in 50PEG electrode can harvest the high reversible capacities of 358.8, 298.6, 253.2, 220.6, 192.5, 170.3, 148.5, and 95.5 mAh g^−1^ at 0.5, 1, 2, 4, 6, 8, 10, and 20 A g^−1^, respectively, and the specific capacities can be fully recovered when the applied current density turns back. Moreover, the corresponding discharge–charge curve (Fig. 3b–c) also confirms the well-retained voltage plateau of electrode in modified electrolyte even at the ultrahigh current density. Note that such storage capability is also superior to many other kinds of cathode materials reported previously (Fig. 3d). While for the storage reversibility, the cycling performance of V_2_O_3_/C electrode was tested at 0.5, 5, and 20 A g^−1^, respectively. Although the electrode in 0PEG electrolyte enables delivering a higher specific capacity of 473.2 mAh g^−1^ in the initial cycle at 0.5 A g^−1^; however, it would quickly decay to 175.3 mAh g^−1^ after 200 cycles (Fig. S5). In contrast, although the electrode in the 50PEG electrolyte would also undergo an activation process during initial few cycles, it can maintain a high discharge capacity of 341.1 mAh g^−1^ after 600 cycles. Besides, similar result also can be observed when the current density was improved to 5 A g^−1^, the electrode can steadily work and retain the discharge capacity of 222.5 mAh g^−1^ after 6000 cycles (Fig. 3e). More surprisingly, an excellent cyclic stability at the ultrahigh current density of 20 A g^−1^ also can be realized in the modified electrolyte, even after 18,000 cycles with the retained capacity of 121.8 mAh g^−1^ and nearly 100% coulombic efficiency (Fig. 3f). As described in Fig. 3g, such ultrastable cyclic performance is also significantly higher than that of many previous reports, such as V_2_O_5_·nH_2_O/graphene [36], MoS_2_/graphene [37], LiV_2_(PO_4_)_3_ [38]_,_ and KV_2_O_4_PO_4_·3.2H_2_O [39]. Notably, excepting the V_2_O_3_/C electrode, our results demonstrated that such anti-proton electrolyte is also conducive to improve the storage capability and cyclic stability of V_2_O_5_ (Fig. S6) and VO_2_ electrodes (Fig. S7), implying it as a general modification strategy to develop the high-performance aqueous Zn-vanadium oxide batteries.

### Energy Storage Mechanism of Aqueous Zn-V_2_O_3_/C Battery

**Fig. 4:**
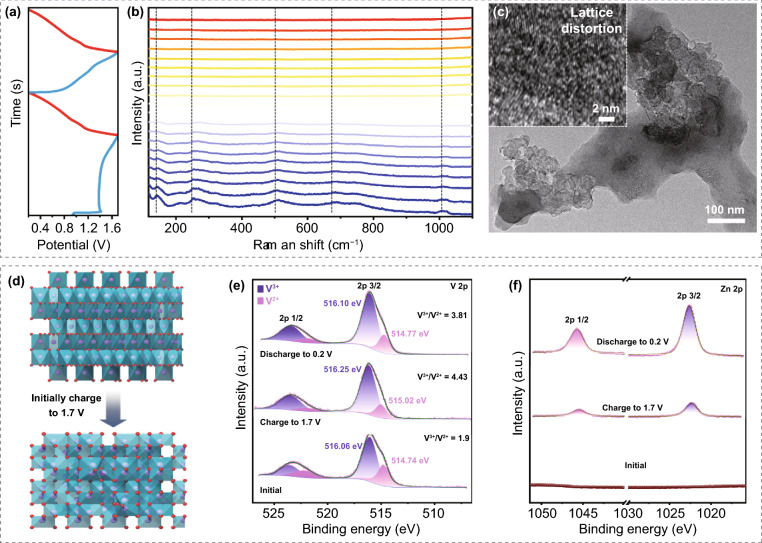
**a** and **b** In-situ Raman spectrum of the initial two cycles for V_2_O_3_/C electrode using the 50% PEG electrolyte. **c** TEM image and HRTEM image (inset) of the V_2_O_3_/C after charged to 1.7 V in the first cycle. **d** Schematic illustration of lattice distortion of V_2_O_3_ after initially charged to 1.7 V. High-resolution XPS spectra of **e** V 2*p* and **f** Zn 2*p* of the V_2_O_3_/C electrode at different state of the first cycle

Next, in order to study the storage mechanism of V_2_O_3_/C electrode and further reveal the influence of such anti-proton electrolyte on tuning the ion storage behavior, the structural evolution of electrode was initially investigated by in-situ electrochemical Raman spectrum (Fig. S8). As shown in Fig. 4a–b, during the charge process of the first cycle, the intensity of all the vibration peaks would gradually weaken and broader. TEM and corresponding HRTEM images (Fig. 4c) reveal the lattice distortion of V_2_O_3_ after initially charged to 1.7 V. It is believed that such lattice distortion (Fig. 4d) would weaken the strong binding of vanadium–oxygen bonds and leading to the above change of vibration peaks [40]. Then, no large change of vibration peaks was observed in the subsequent second cycle (Fig. S9). Excepting that, ex-situ XPS spectra of V 2*p* and Zn 2*p* of V_2_O_3_/C electrode during the first cycle were also investigated. As shown in Fig. 4e, the ratio of V^3+^/V^2+^ would increases from 1.9 (at initial state) to 4.43 when charged to 1.7 V; however, this value only decreases back to 3.81 after subsequently discharged to 0.2 V. Such result implies the irreversible transformation of lattice distortion of V_2_O_3_. While in the high-resolution Zn 2*p* XPS spectra (Fig. 4f), no Zn signal peak was detected at the initial state; however, Zn 2*p*_2/3_ and Zn 2*p*_1/2_ signal peaks would appear after discharged to 0.2 V, this should be mainly ascribed to the insertion of Zn^2+^ into the distorted V_2_O_3_ framework.

**Fig. 5:**
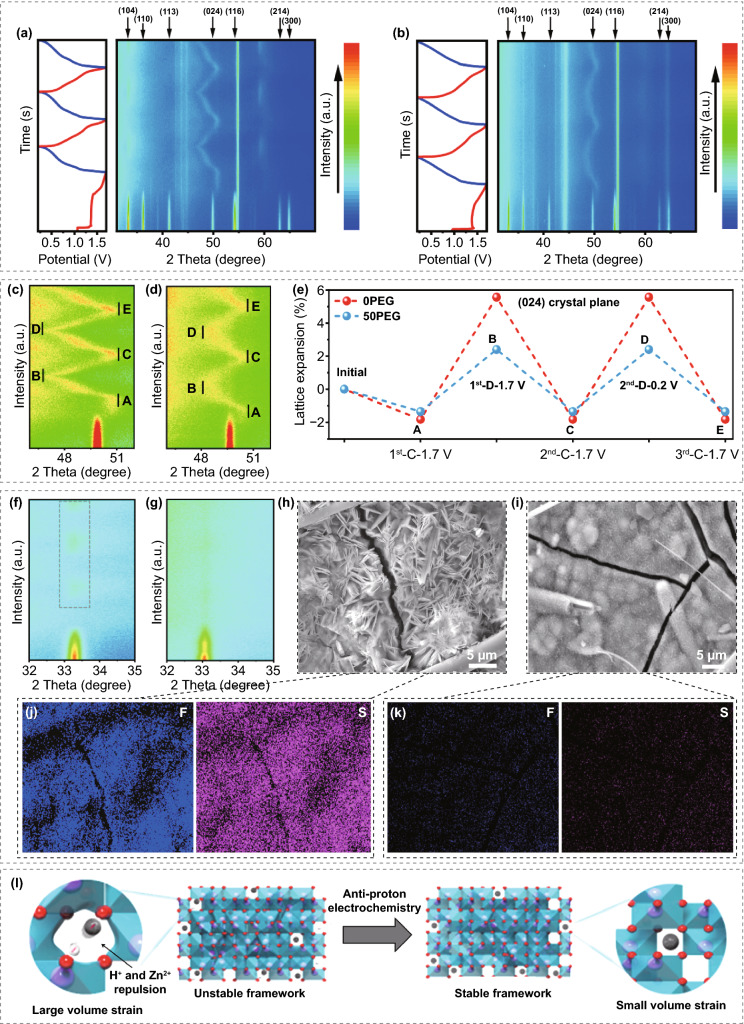
In-situ XRD patterns of V_2_O_3_/C electrode in the first three cycles in **a** 0PEG and **b** 50PEG electrolyte. High-resolution contour maps of in-situ XRD pattern between 46° and 52^o^ in the **c** 0PEG and **d** 50PEG electrolyte. **e** Lattice-expand ratio evolution of (024) plane derived from the in-situ XRD data. High-resolution contour maps of in-situ XRD pattern between 32° and 35^o^ in the **f** 0PEG and **g** 50PEG electrolyte. FESEM image and corresponding EDS elemental mapping of V_2_O_3_/C electrode after 200 cycles at the current density of 0.5 A g^−1^ in (**h**, **j**) 0PEG and (**i**, **k**) 50PEG electrolyte. **l** The evolution of storage mechanism of V_2_O_3_ host modified by the anti-proton electrolyte

In-situ XRD technique (Fig. S10) was also adopted to further investigate the phase evolution of V_2_O_3_/C electrode. In detail, Fig. 5a–b depicts the in-situ XRD patterns of V_2_O_3_/C electrode in 0PEG and 50 PEG electrolyte of the first three cycles. Before cycling, several strong diffraction peaks located at 32.97°, 35.95°, 41.01°, 49.58°, 53.85°, 62.75°, and 64.63° can be detected, which should be corresponding to the (104), (110), (113), (024), (116), (214), and (300) crystal plane of rhombohedral V_2_O_3_ phase, respectively [33]. However, during the charge process in the first cycle, all those diffraction peaks would gradually weaken, and even completely disappear till charged to 1.7 V. Note that only the (024) plane was shifted to the right (point A in Fig. 5c–d) in both two kinds of electrolyte, this process should be attributed to the lattice distortion of V_2_O_3_, combining with the above in-situ Raman and HRTEM characterizations [40]. With the following discharge process continues, the (024) crystal plane would gradually shift to the left point (B), corresponding to the lattice expansion owing to the ions intercalation. Moreover, a high reversibility of ions intercalation/extraction can be achieved in the subsequent two cycles. Interestingly, it also can be found that the degree of lattice expansion in 0PEG electrolyte is much larger than that in 50PEG electrolyte, as shown in Fig. 5e. In detail, the lattice expansion ratio of (024) crystal plane reaches 5.57 when discharged to 0.2 V in 0PEG electrolyte, much larger than that of 2.40 in 50PEG electrolyte. It is no doubt that a large lattice expansion is not conducive to structural stability [41, 42]. In addition, an impurity peak located at 33.31° also can be observed in 0PEG electrolyte system (Fig. 5f), but cannot find in 50PEG electrolyte (Fig. 5g). According to the previous reports, this diffraction peak should be ascribed to the alkali-type by-product of Zn_x_(OTf)_y_(OH)_2x−y_·nH_2_O [43]. Note that the formation of such side reaction should be originated from the dramatical increasing concentration of OH^−^ near the cathode–electrolyte interface owing to the H^+^ insertion into the host [44]. Besides, the FESEM image of V_2_O_3_/C electrodes after 200 cycles at 0.5 A g^−1^ also shows large amount of flaky precipitate attached to the surface of electrode in 0PEG electrolyte (Fig. 5h). Corresponding EDS mapping (Figs. 5j and S11) reveals the strong signal of F and S elements, further confirming it is the Zn_x_(CF_3_SO_3_)_y_(OH)_2x−y_·nH_2_O [45, 46]. On the contrary, a relative clean surface without obvious formation of flaky by-product can be seen for the cycled V_2_O_3_/C electrode in 50PEG electrolyte (Figs. 5i, k and S12). Therefore, it is demonstrated that the H^+^ intercalation behavior can be significantly inhibited in 50PEG electrolyte. Accordingly, the formation of by-product also can be effectively alleviated. Indeed, both of the in-situ XRD research and ex-situ FESEM characterizations are in good agreement with the result of MD simulation. To demonstrate the possibility of such anti-proton electrolyte as universal strategy, in-situ XRD investigations of VO_2_ electrode (Figs. S13–S15) and V_2_O_5_ electrode (Figs. S16–S18) in both 0PEG and 50PEG electrolyte were also carried out. Interestingly, similar results of smaller lattice expansion and less by-product formation also can be achieved in the VO_2_ and V_2_O_5_ case when applied the modified electrolyte. This can be explained the significant improvement of their cyclic stability in 50PEG electrolyte in the section of electrochemical performance test. As depicted in Fig. 5l, benefited from the anti-proton electrolyte, the Zn^2+^ and H^+^ co-intercalation mechanism of V_2_O_3_/C electrode in acidic electrolyte can be regulated to the Zn^2+^-dominated intercalation mechanism. In fact, although the dual ions insertion can bring a higher specific capacity, the inhibition of proton insertion could avoid the large repulsion between H^+^ and Zn^2+^, which usually leads to the large lattice expansion and damage the host framework. Besides, the formation of non-electrochemically active by-product in the cathode–electrolyte interface would also hinder the interfacial transport kinetics, which will be discussed in the next section.

### Transport Kinetics Investigation of Aqueous Zn-V_2_O_3_/C Battery

**Fig. 6:**
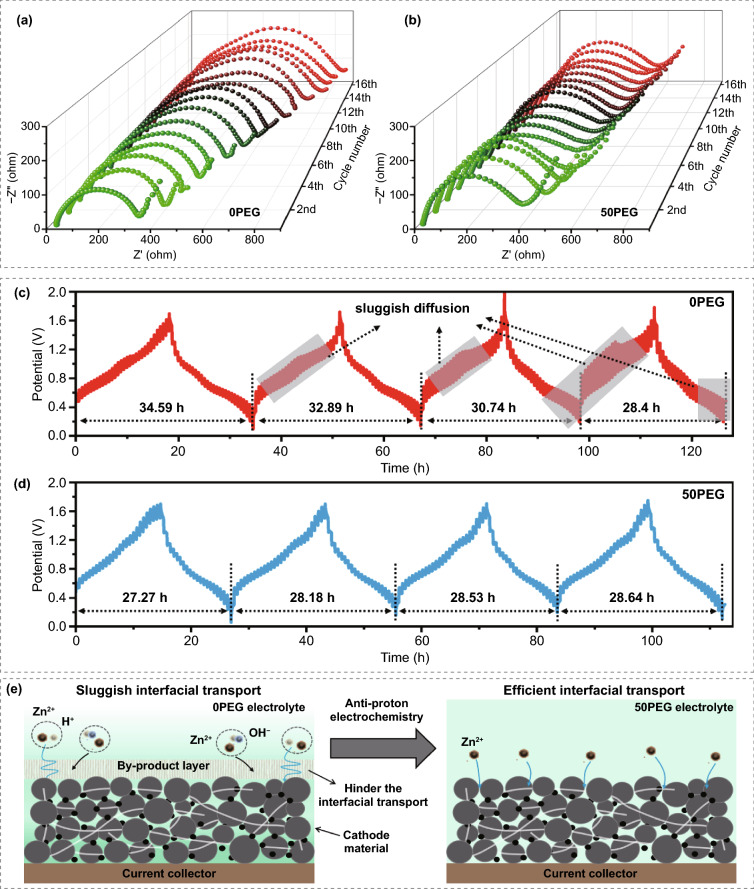
In-situ electrochemical impedance spectroscopy (EIS) test of the V_2_O_3_/C electrode in **a** 0PEG and **b** 50PEG electrolyte during the initial 16 cycles, at the current density of 0.5 A g^−1^. The Galvanostatic Intermittent Titration Technique (GITT) of V_2_O_3_/C electrode cycled in **c** 0PEG electrolyte and **d** 50PEG electrolyte with a 0.2 A g^−1^ pulse current density, 5 min pulse time and 30 min relaxation time. **e** The evolution of interfacial transport kinetics of electrode regulated by the anti-proton electrolyte

Since the by-product formation in the cathode–electrolyte interface would greatly influence the ion transport and charge transfer, it is also necessary to clarify the influence of such anti-proton strategy on the transport kinetics of electrode. To demonstrate that, in-situ electrochemical impedance spectroscopy (EIS) and galvanostatic intermittent titration technique (GITT) were performed to explore the evolution of transport kinetics of electrode during cycling. On one hand, Fig. 6a–b shows the EIS plots of V_2_O_3_/C electrode in 0PEG and 50PEG electrolyte during cycling. Note that all the Nyquist plots consist of a semicircle in the high frequency region and a linear part in the low-frequency region, where the semicircle region represents the charge transfer impedance (*R*_ct_). Obviously, the *R*_ct_ in the 0PEG electrolyte system shows a gradual increasing trend along with the cycling proceeds. Such result should be resulted from the generation of non-electrochemically active and low electronic conductive by-product in the cathode–electrolyte interface, which not only consumes the Zn^2+^ of electrolyte but also impedes the efficient ion/electron transport during cycling. In sharp contrast to that, in 50PEG electrolyte system, only a slight increment of *R*_ct_ in the initial two cycles, and it can retain stable in the subsequent cycles, representing it an efficient and stable interfacial transport kinetics. As shown in Fig. 6c, the GITT curve of V_2_O_3_/C electrode in 0PEG electrolyte indicates that the specific capacity of battery continues to decline and even appear the overvoltage region during charge and discharge process, as marked in the shaded area. Such overvoltage region usually represents a sluggish ion diffusion, which perhaps due to the spontaneous pre-intercalation of H^+^ or competitive intercalation of H^+^/Zn^2+^, and the generation of by-product in the cathode–electrolyte interface [47, 48]. Differently, the electrode in 50PEG electrolyte (Fig. 6d) shows a stable specific capacity without any overvoltage region. Corresponding zinc-ion diffusion coefficient (D_Zn_^2+^) is calculated and shown in Fig. S19. Compared with the D_Zn_^2+^ stabilize in the range of 10^−10^–10^−11^ cm^2^ s^−1^ achieved in the 50PEG electrolyte, additionally, there exhibits ultralong relaxation region in the 0PEG electrolyte, with a lower ion diffusion coefficient of 10^−11^–10^−13^ cm^2^ s^−1^. Therefore, combined with the results of in-situ EIS and GITT studies, we demonstrate that the formation of by-product would leads to the sluggish interfacial ion/electron transport, as illustrated in Fig. 6e. Such adverse effect will further reduce the utilization of active material and thus sharply attenuating the rate performance and cyclic stability of electrode. Fortunately, all those issues can be effectively addressed through anti-proton electrochemistry, and a fast storage kinetics also can be achieved (Fig. S20).

### Electrochemical Performance of Zn||Zn Symmetric Cell

**Fig. 7:**
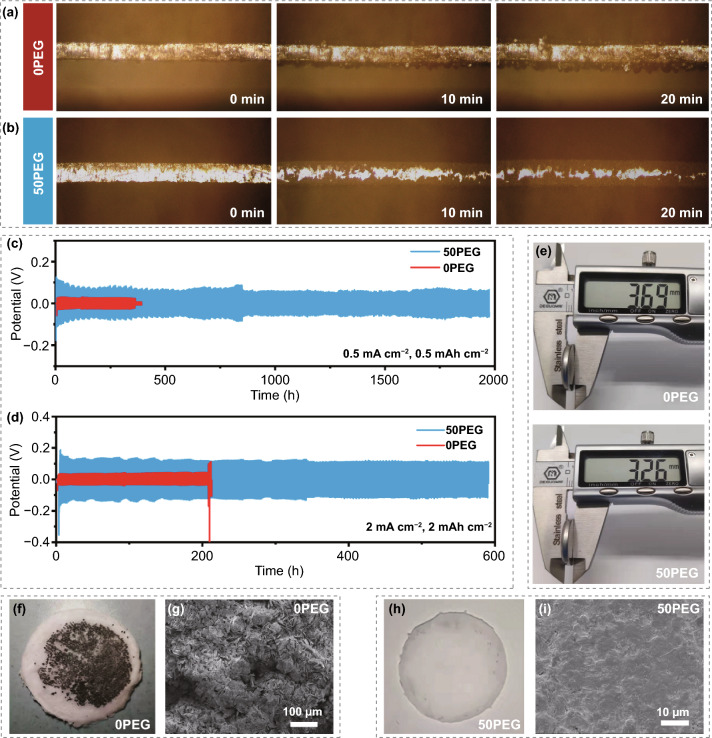
In-situ optical observation of Zn deposition in **a** 0PEG and **b** 50PEG electrolyte at the current density of 10 mA cm^−2^. The cyclic stability of Zn||Zn symmetric cell at the testing conditions of **c** 0.5 mA cm^−2^, 0.5 mAh cm^−2^ and **d** 2 mA cm^−2^, 2 mAh cm^−2^, respectively. **e** The thickness of Zn||Zn symmetric cell after cycling for 200 h at 2 mA cm^−2^ and 2 mAh cm^−2^, and the digital images of corresponding GF separator and FESEM images of corresponding Zn metal anodes in (**f**, **g**) 0PEG and (**h**, **i**) 50PEG electrolyte

Since Zn metal anode also plays a critical role in influencing the electrochemical performance of AZIBs, herein, the influence of such anti-proton electrolyte on regulating the Zn plating/stripping behavior and HER phenomenon are also investigated. In-situ optical microscopy (Fig. S21) was adopted to reveal the morphology evolution of Zn metal anode upon continue deposition. When in the 0PEG electrolyte (Fig. 7a), rapid growth of dendrite on the metal anode surface can be clearly observed along with the deposition extending to 20 min. On the contrary, a smooth and dense deposition surface can be achieved in the 50PEG electrolyte even after 20 min (Fig. 7b). Such result indicates that the PEG molecular chain is beneficial to guide the uniform Zn deposition. Then, the cyclic stability of Zn||Zn symmetric cell was tested. Unlike the rapid short circuit in the 0PEG electrolyte, the reversibility of Zn plating/stripping can be significantly enhanced in the 50 PEG electrolyte. In detail, the cell can steadily work for nearly 2000 and 600 h at the testing conditions of 0.5 mA cm^−2^, 0.5 mAh cm^−2^ (Fig. 7c) and 2 mA cm^−2^, 2 mAh cm^−2^ (Fig. 7d), respectively, compared with short circuit after cycling for 354 and 210 h for the unmodified cell. Besides, taking the initial thickness of assembled cell (3.21 mm, Fig. S22) as the reference, the thickness of cell with 0PEG electrolyte would dramatically increase to 3.69 mm after cycling for 200 h at 2 mA cm^−2^ and 2 mAh cm^−2^; however, only a minor change was observed for the modified cell (3.26 mm, Fig. 7e). Such swollen phenomenon should be mainly due to internal expansion caused by hydrogen evolution [49]. Meanwhile, the digital images of corresponding GF separator and FESEM images of cycled Zn metal anode further demonstrate the dendrite growth and even piercing separator in the 0PEG electrolyte (Fig. 7f–g). Unlike that, smooth deposition without obvious dendrite formation can be observed in the 50PEG electrolyte case (Fig. 7h and i). Subsequently, the characterizations of XRD pattern (Fig. S23) and EDS elemental mapping (Fig. S24) also confirm the function of inhibiting side reactions by the anti-proton electrolyte. Therefore, in regard to the Zn metal anode side, we demonstrate that such anti-proton electrolyte is beneficial to not only guide the uniform Zn deposition, but also inhibit the HER and by-product generation.

## Conclusion

**Scheme 2:**
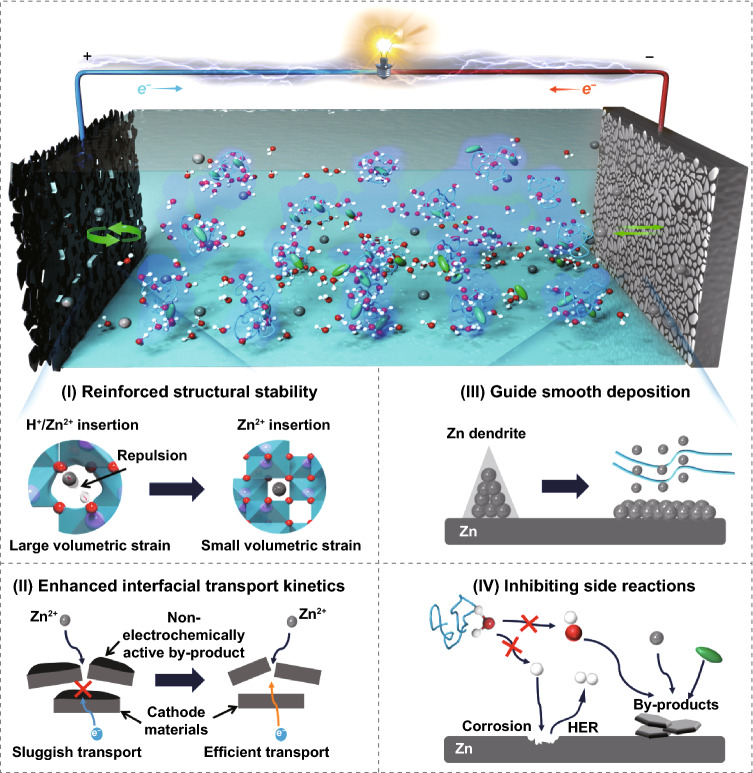
Illustration of integrated synergetic modification mechanism of aqueous Zn-vanadium oxide battery enabled by multifunctional anti-proton electrolyte

In summary, in order to simultaneously address the system problems that cause the electrochemical failure of zinc-vanadium oxide battery, we propose the strategy of anti-proton electrolyte as the “all-in-one” strategy for designing high-performance aqueous zinc-vanadium oxide battery. The investigations of molecular dynamics simulation and experiments indicate that the PEG 400 additive can not only regulate the solvation structure of Zn^2+^ but also suppress the ionization of free water molecules. Then, take the V_2_O_3_/C cathode as research target, coupling with the in-situ XRD and in-situ EIS studies, we demonstrate that such anti-proton electrolyte enables inhibiting the H^+^ insertion and corresponding associated side reactions, thus realizing the small lattice expansion of V_2_O_3_ host and stable interfacial ion/electron transport of electrode. Besides that, in regarding to the Zn metal anode, in-situ optical observation and ex-situ structural characterizations further confirm that such anti-proton electrolyte is conductive to guide the uniform Zn deposition and inhibit the HER. As a result, benefited from the integrated synergetic modification mechanism of such multifunctional anti-proton electrolyte (Scheme 2), the as-assembled Zn-V_2_O_3_/C battery possesses a significantly enhanced rate performance, which can deliver the reversible capacities of 358.8, 298.6, 253.2, 220.6, 192.5, 170.3, 148.5, and 95.5 mAh g^−1^ at the current density of 0.5, 1, 2, 4, 6, 8, 10, and 20 A g^−1^, respectively. Representatively, at the ultrahigh current density of 20 A g^−1^, such modified battery can retain a high specific capacity of 121.8 mAh g^−1^ even after 18,000 cycles with a nearly 100% coulombic efficiency, showing an ultrastable cycle reversibility. This research uncovers a brand-new integrated synergetic modification mechanism of aqueous Zn-vanadium oxide battery, which is highly expected to lay a foundation for developing high-performance AZIBs.

## Supplementary Information

Below is the link to the electronic supplementary material.

Supplementary file1 (PDF 2478 kb)
