# Supplementary material for: A Multifunctional Anti-Proton Electrolyte for High-Rate and Super-Stable Aqueous Zn-Vanadium Oxide Battery
Source: Nanomicro Lett. 2022 Aug 2;14:154. doi: 10.1007/s40820-022-00907-4 (PMC9346040; doi:10.1007/s40820-022-00907-4)
Supplement: Supplementary file 1 — Supplementary file1 (PDF 2478 kb) [file 40820_2022_907_MOESM1_ESM.pdf]

Supporting Information for

A Multifunctional Anti-Proton Electrolyte for High-Rate and Super-Stable Aqueous Zn-Vanadium Oxide Battery

Yangwu Chen[1], Dingtao Ma[1, 2, *], Kefeng Ouyang[1], Ming Yang[1], Sicheng Shen[1], Yanyi Wang[1], Hongwei Mi[1], Lingna Sun[1], Chuanxin He[1], Peixin Zhang[1, *]

[1]College of Chemistry and Environmental Engineering, Shenzhen University, Shenzhen 518060, P. R. China

[2]Institute of Microscale Optoelectronics, Shenzhen University, Shenzhen 518060, P. R. China

*Corresponding authors. E-mail: mdt2500@szu.edu.cn (D.T. Ma), pxzhang@szu.edu.cn (P.X. Zhang)

Supplementary Figures and Tables

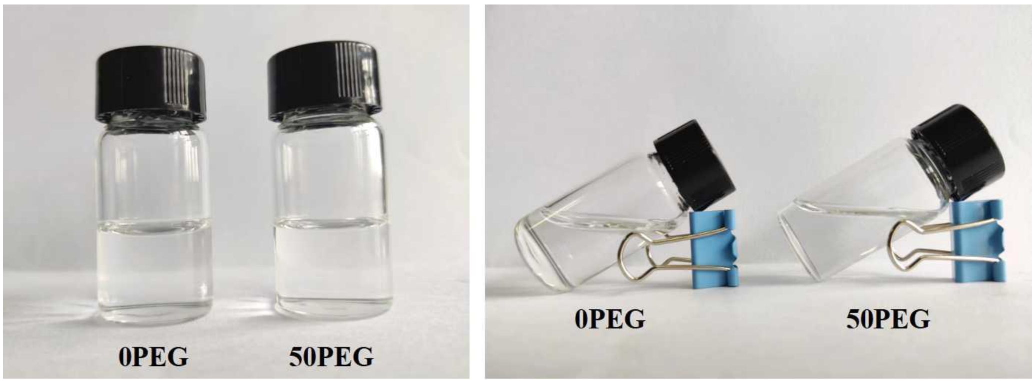

Fig. S1 Digital images of the as-prepared 0PEG and 50PEG electrolyte

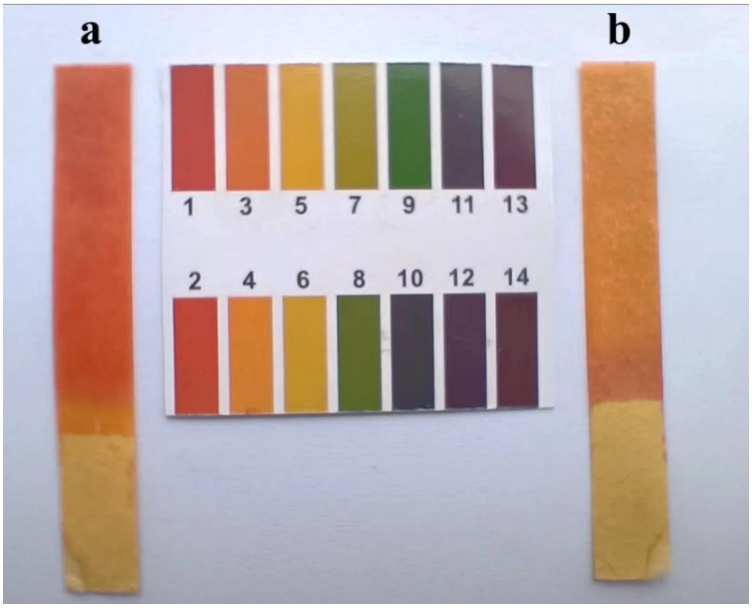

Fig. S2 Digital image of the pH strips after immersion in **a** 0PEG and **b** 50PEG electrolyte

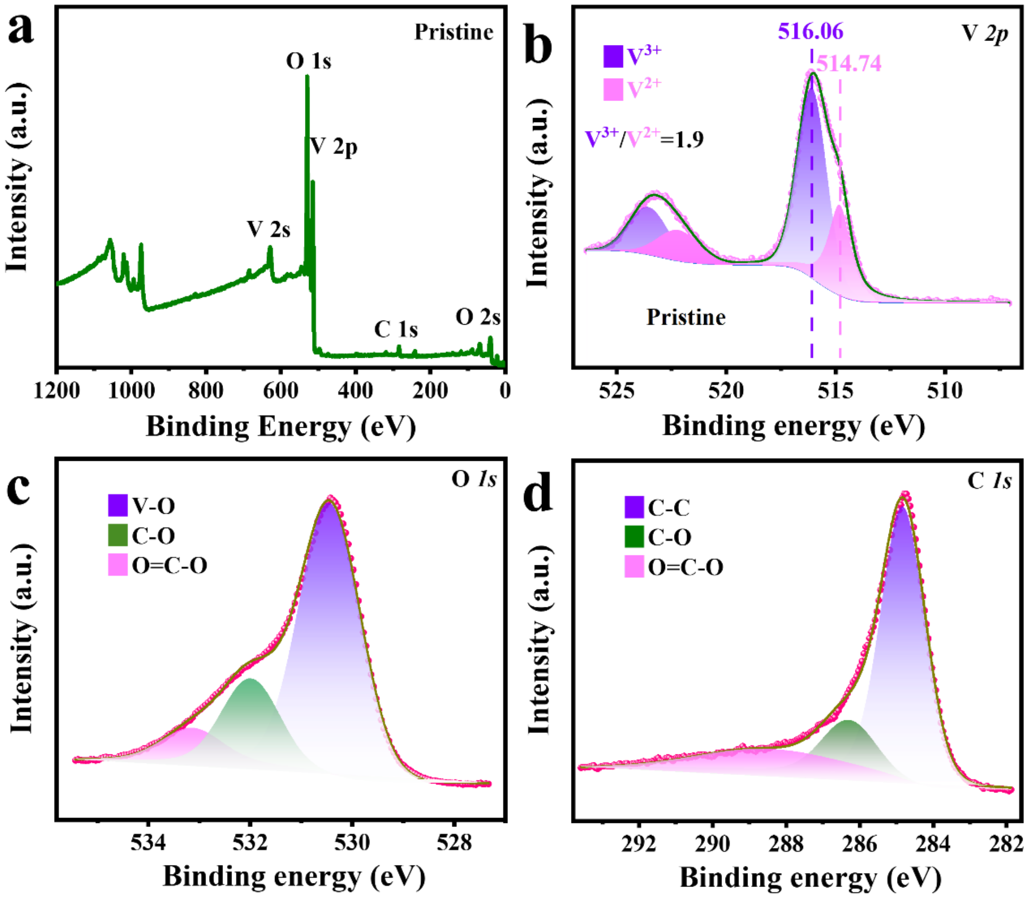

Fig. S3 (**a**) Full XPS spectrum of V_2O_3/C nanosheets. High-resolution XPS spectra of (**b**) V 2p, (**c**) O 1s and (**d**) C 1s of V_2O_3/C nanosheets

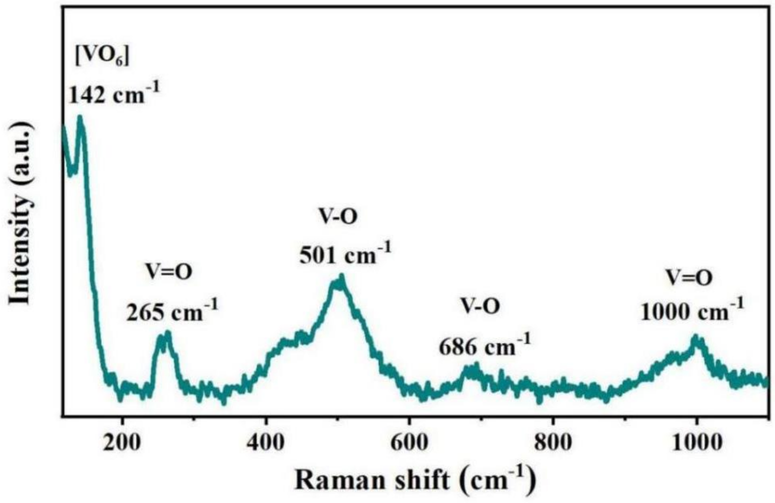

Fig. S4 Raman spectrum of V_2O_3/C nanosheets

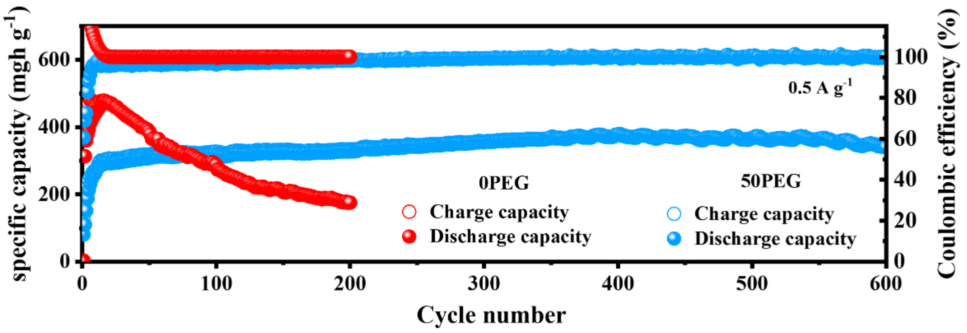

Fig. S5 The cycling performance of V_2O_3/C electrode in the 0PEG and 50PEG electrolyte at the current density of 0.5 A g^{-1}

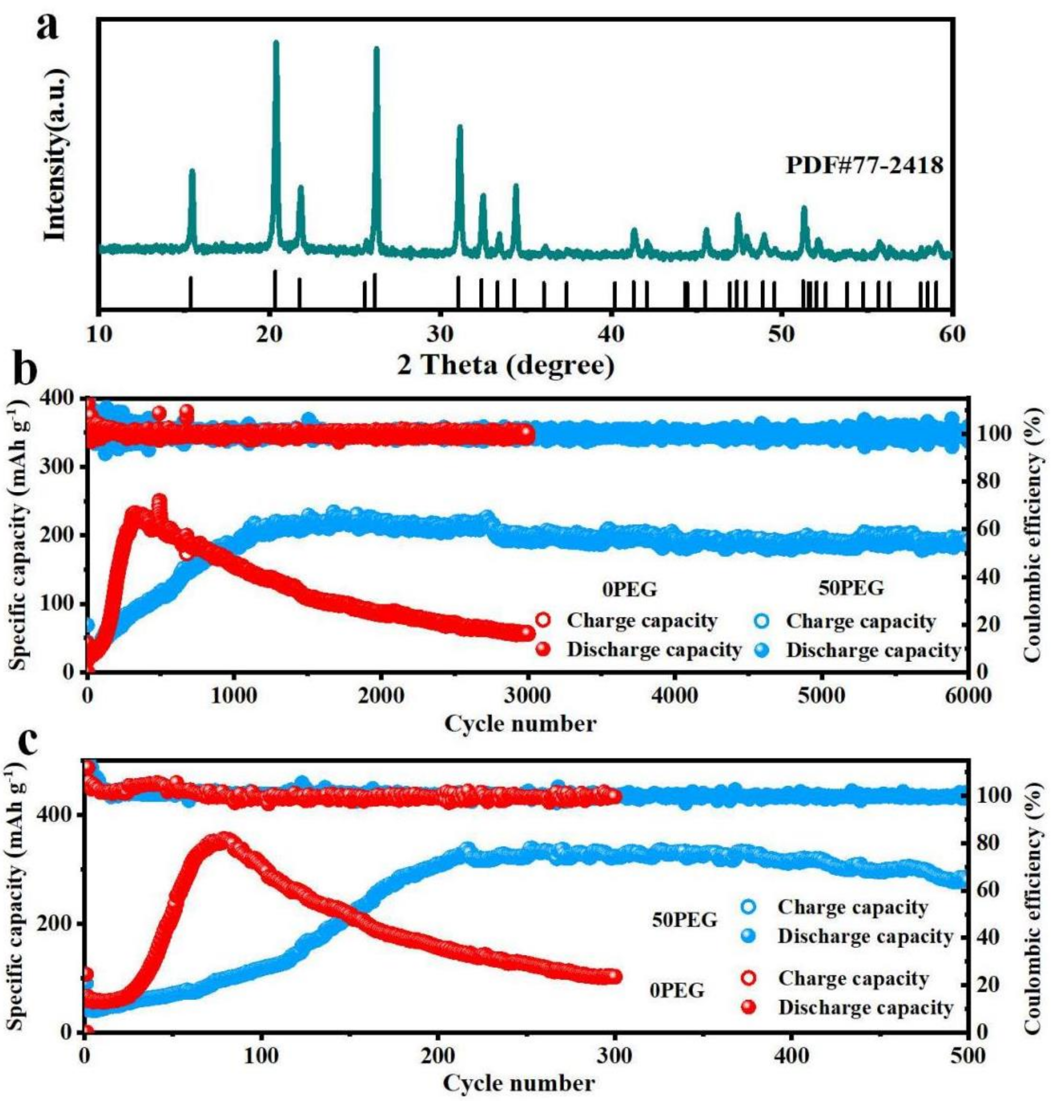

Fig. S6 (**a**) XRD patterns of the commercial V_2O_5 cathode. The cycling performance of the V_2O_5 electrode in the 0PEG and 50PEG electrolyte at the current density of (**b**) 0.5 A g^{-1} and (**c**) 5 A g^{-1}

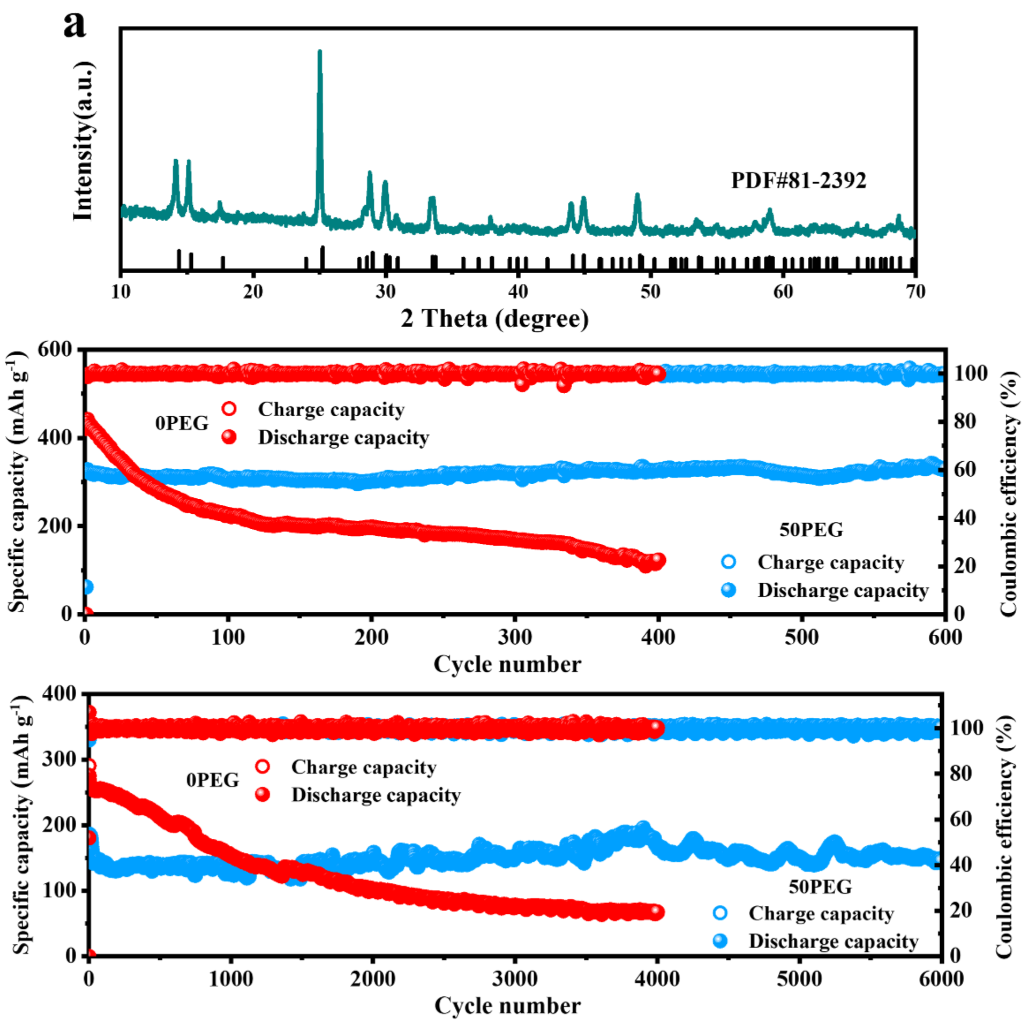

Fig. S7 (**a**) XRD patterns of the as-prepared VO_2 cathode. The cycling performance of the VO_2 electrode in the 0PEG and 50PEG electrolyte at the current density of (**b**) 0.5 A g^{-1} and (**c**) 5 A g^{-1}, respectively

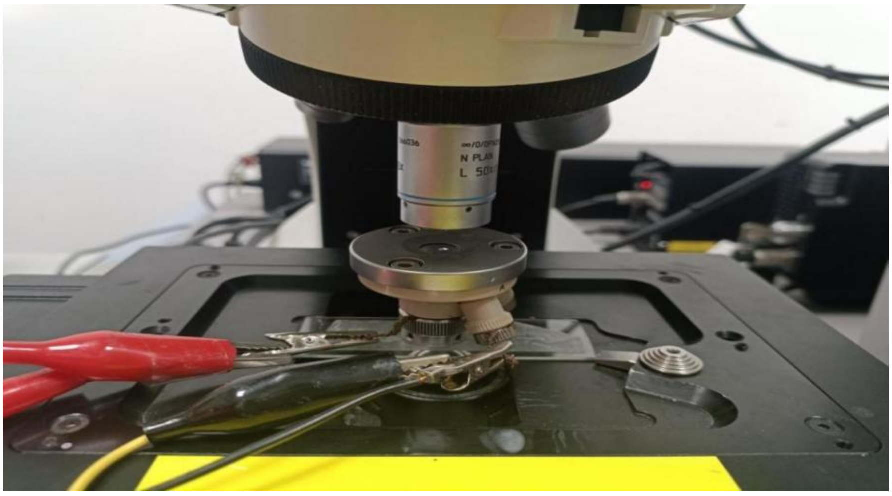

Fig. S8 Digital image of in-situ electrochemical Raman spectroscopy device

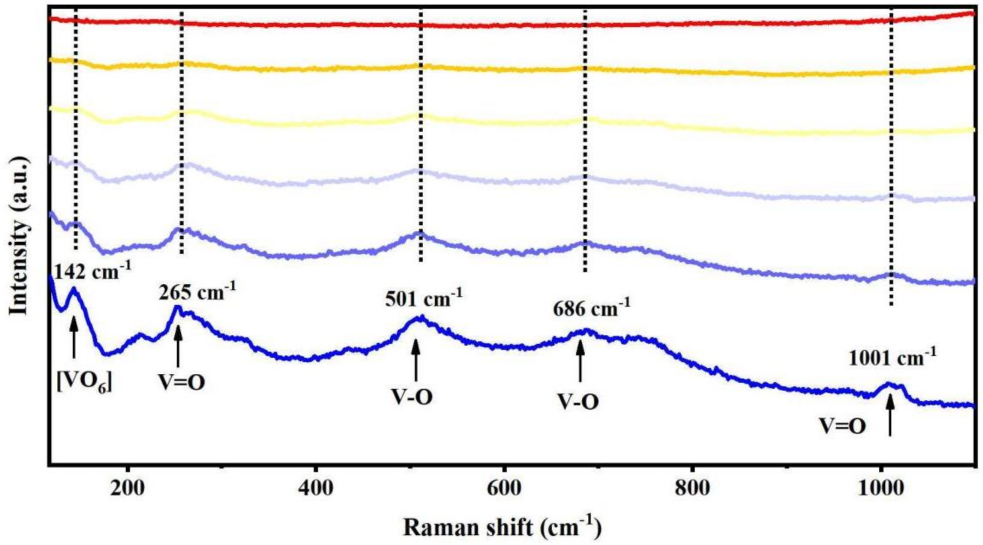

Fig. S9 The corresponding enlarged spectra in Fig. 4b

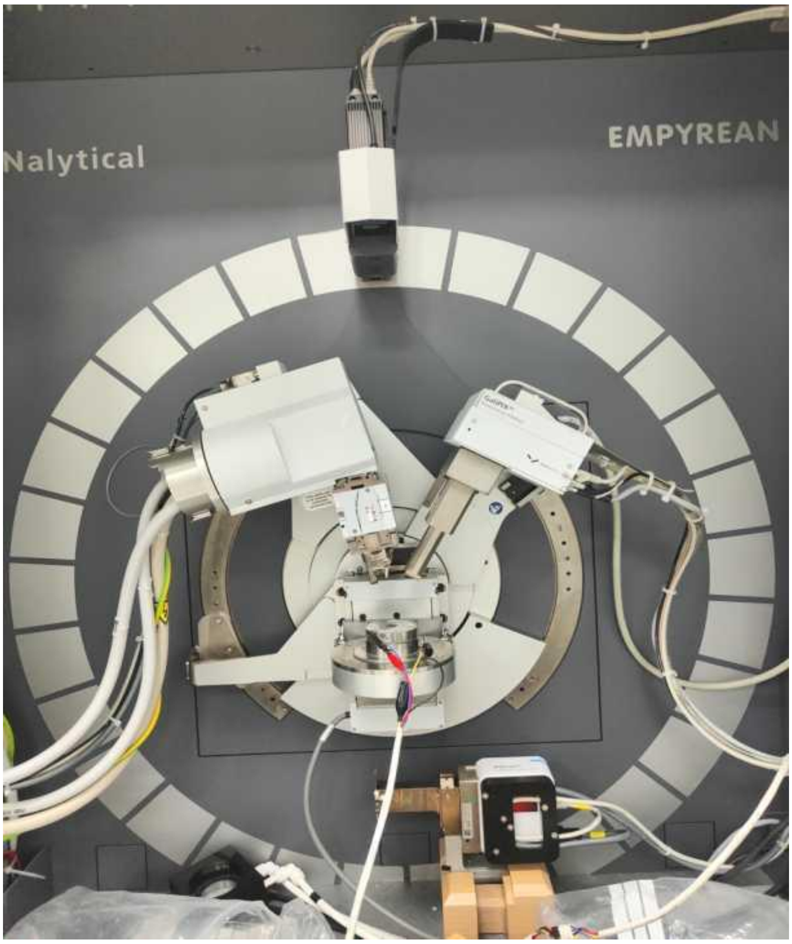

Fig. S10 Digital image of in-situ XRD device

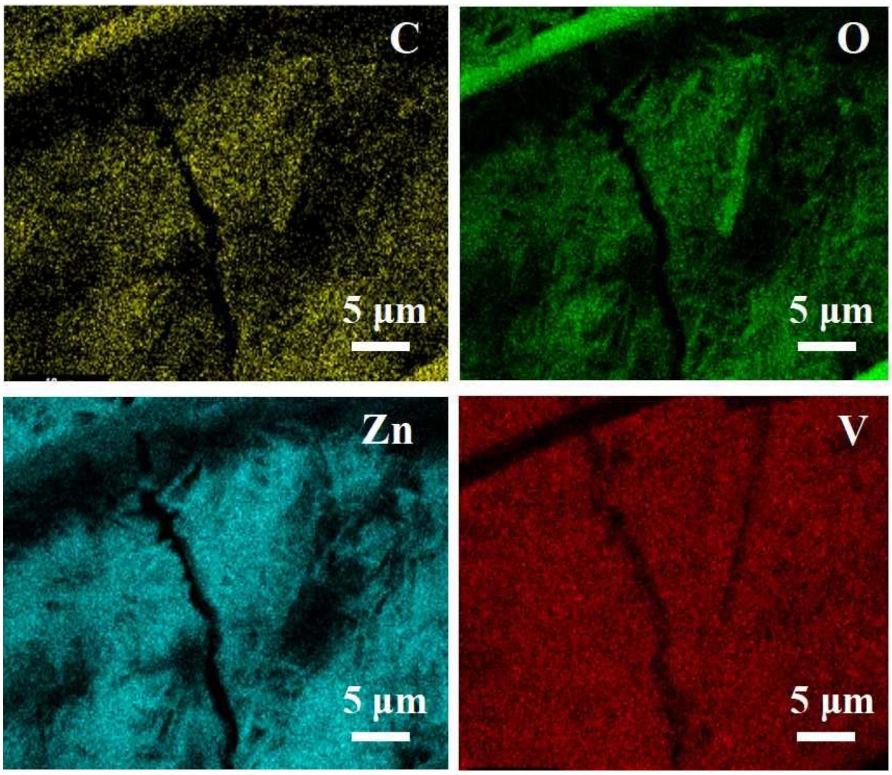

Fig. S11 EDS elemental mapping of V_2O_3/C electrode after 200 cycles at the current density of 0.5 A g^{-1} in the 0PEG electrolyte

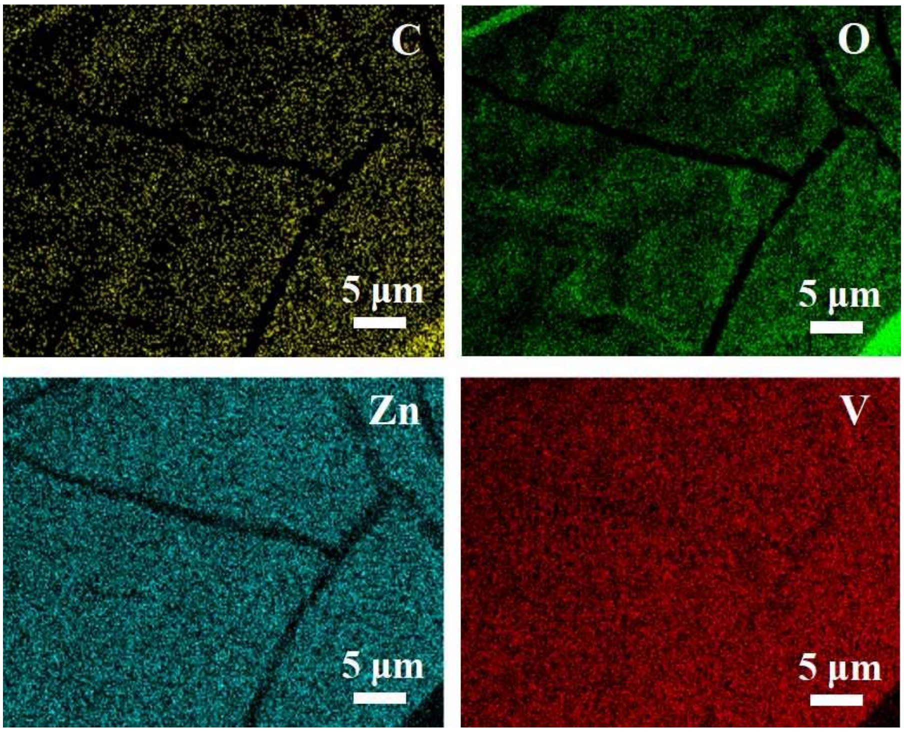

Fig. S12 EDS elemental mapping of V_2O_3/C electrode after 200 cycles at the current density of 0.5 A g^{-1} in the 50PEG electrolyte

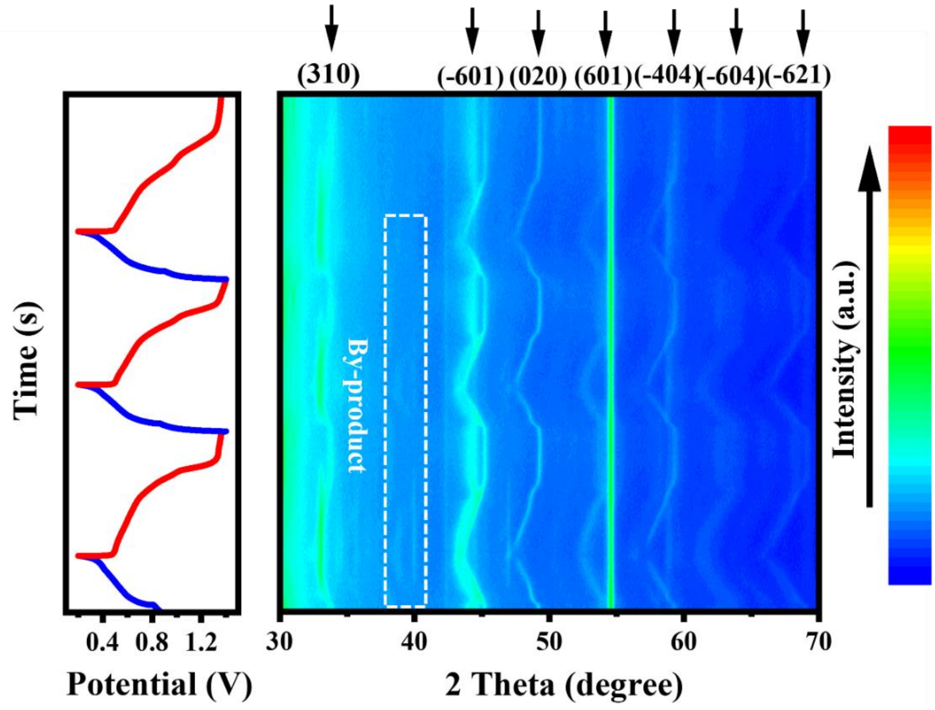

Fig. S13 In-situ XRD analysis of VO_2 in 0PEG electrolyte with a current of 0.3 mA and voltage window of 0.4-1.4 V from 1st to the 3rd cycles

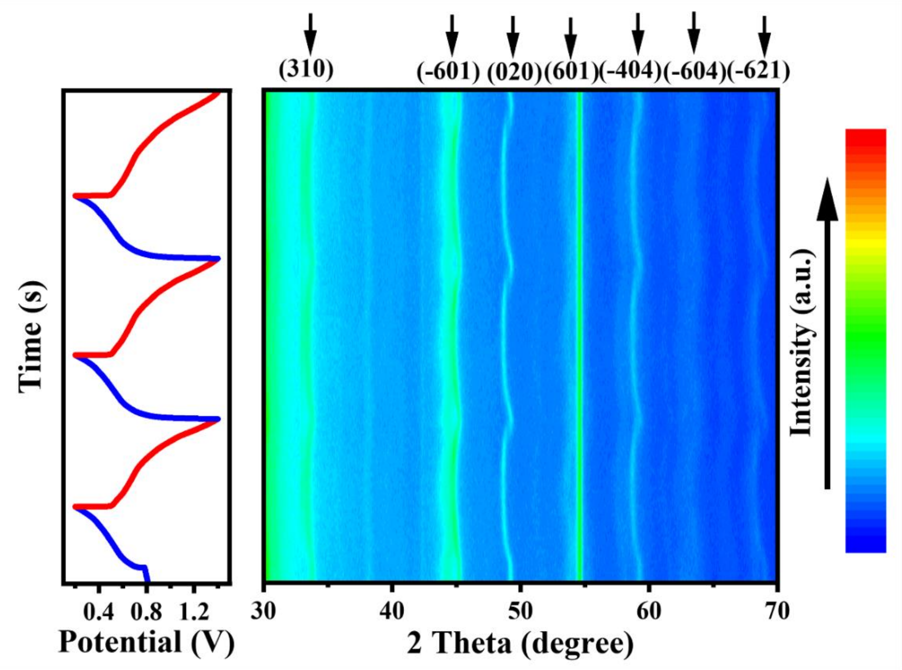

Fig. S14 In-situ XRD analysis of VO_2 in 50PEG electrolyte with a current of 0.3 mA and voltage window of 0.4-1.4 V from 1st to the 3rd cycles

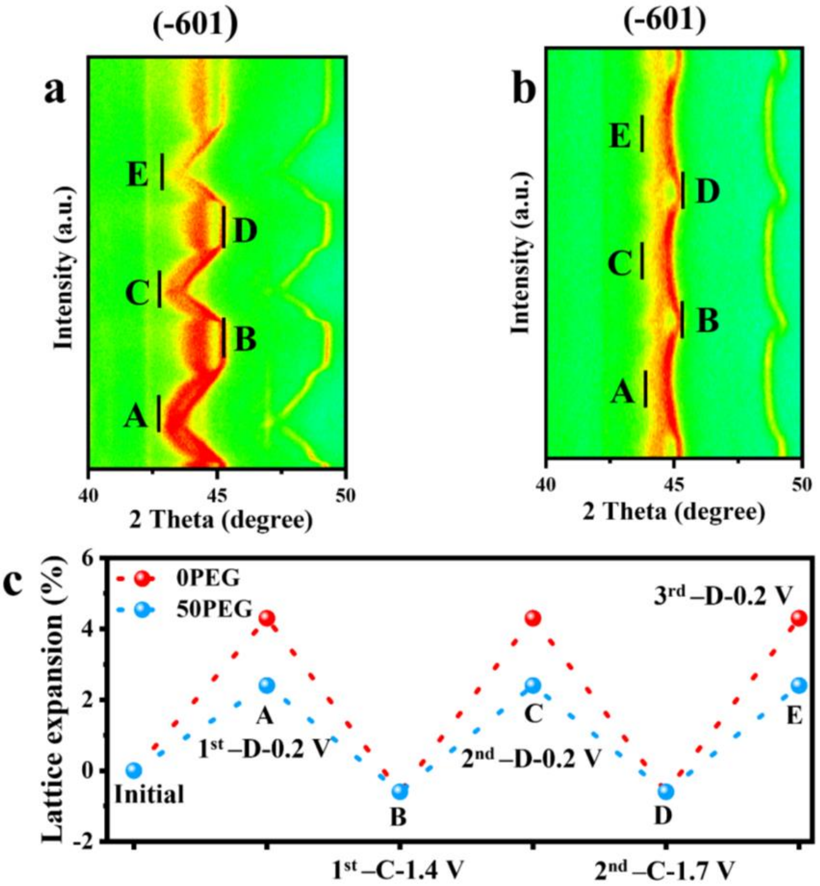

Fig. S15 In-situ XRD analysis of the (-601) plane of VO_2 in (**a**) 0PEG and (**b**) 50PEG electrolytes. (**c**) Lattice-expand ratio evolution derived from In-situ XRD of (-601) plane

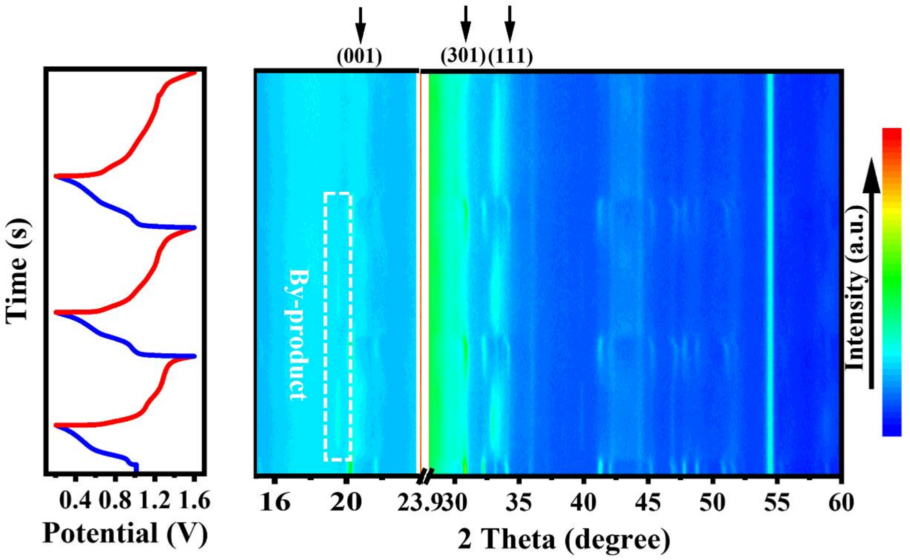

Fig. S16 In-situ XRD analysis of V_2O_5 in 0PEG electrolyte with a current of 0.3 mA and voltage window of 0.2-1.6 V from 1st to the 3rd cycles

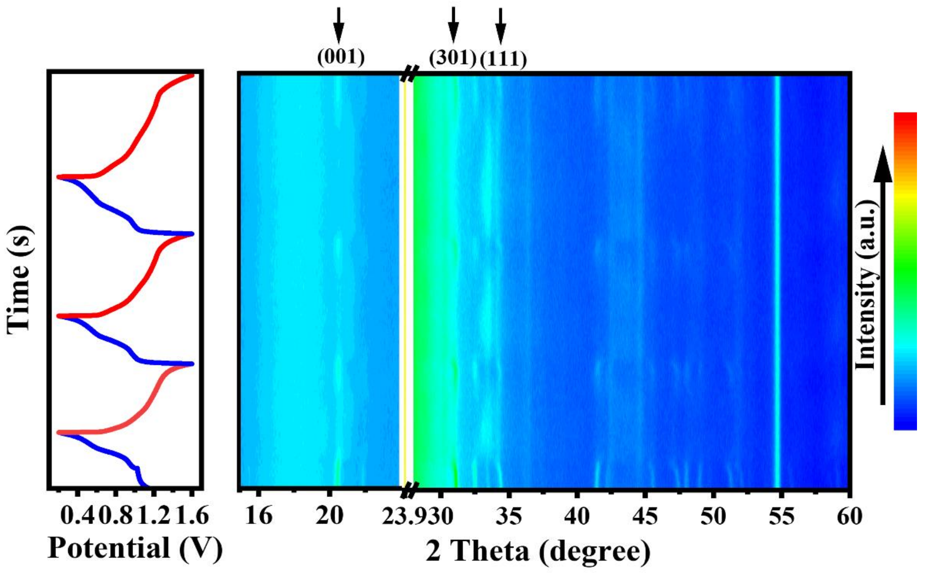

Fig. S17 In-situ XRD analysis of V_2O_5 in 50PEG electrolyte with a current of 0.3 mA and voltage window of 0.2-1.6 V from 1st to the 3rd cycles

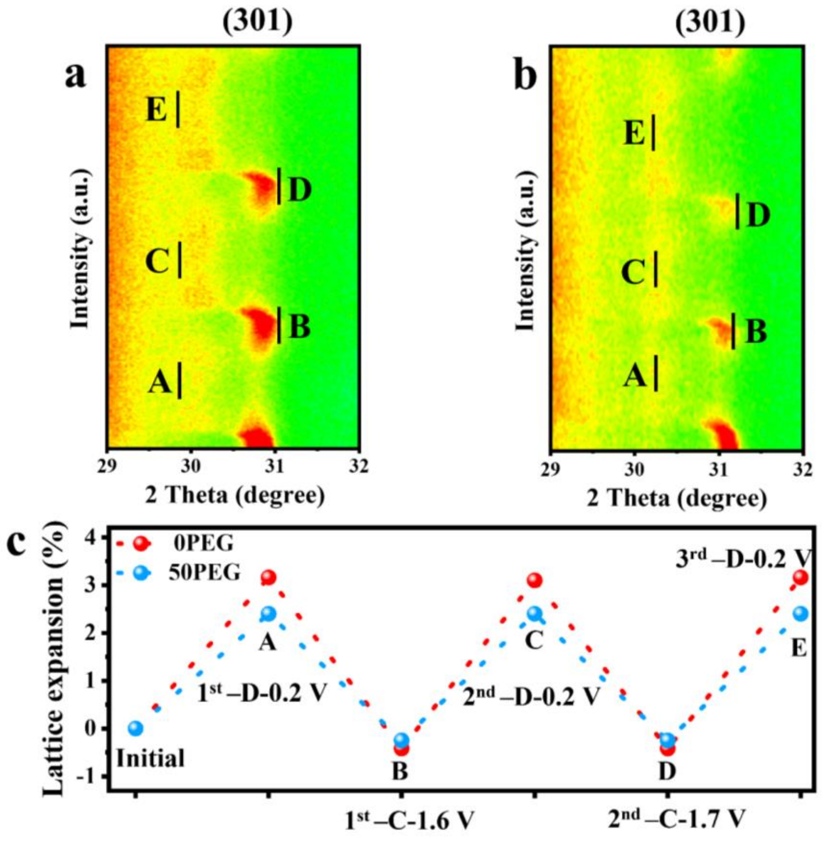

Fig. S18 In-situ XRD analysis of the (301) plane of V_2O_5 in (**a**) 0PEG and (**b**) 50PEG electrolytes. (**c**) Lattice-expand ratio evolution derived from In-situ XRD of (301) plane

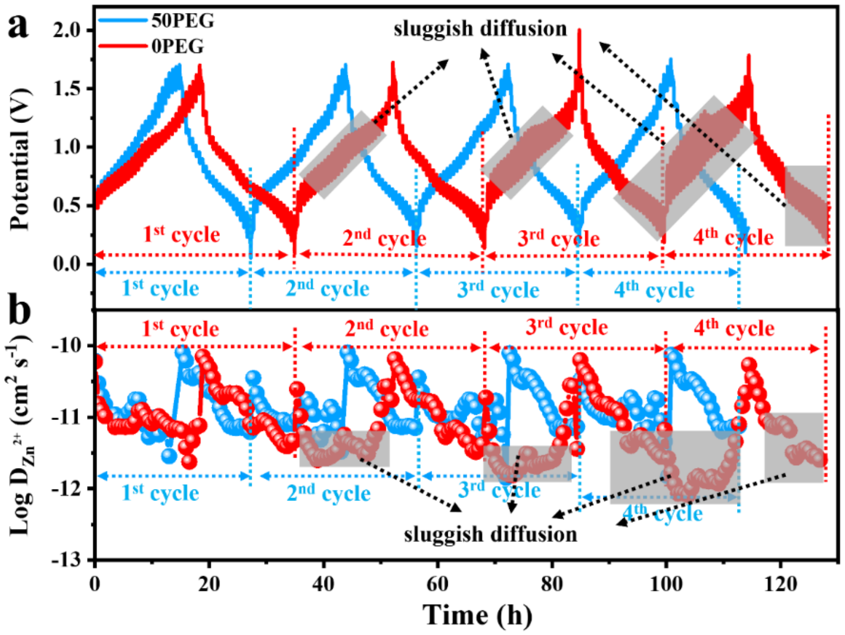

Fig. S19 (**a**) The comparison of galvanostatic Intermittent Titration Technique (GITT) and (**b**) corresponding zinc-ion diffusion coefficient ($D_{Zn}{}^{2+}$) of V_2O_3/C electrode cycled in 0PEG and 50PEG electrolyte at a pulse current density of 0.2 A g $^{-1}$, 5 min pulse time and 30 min relaxation time

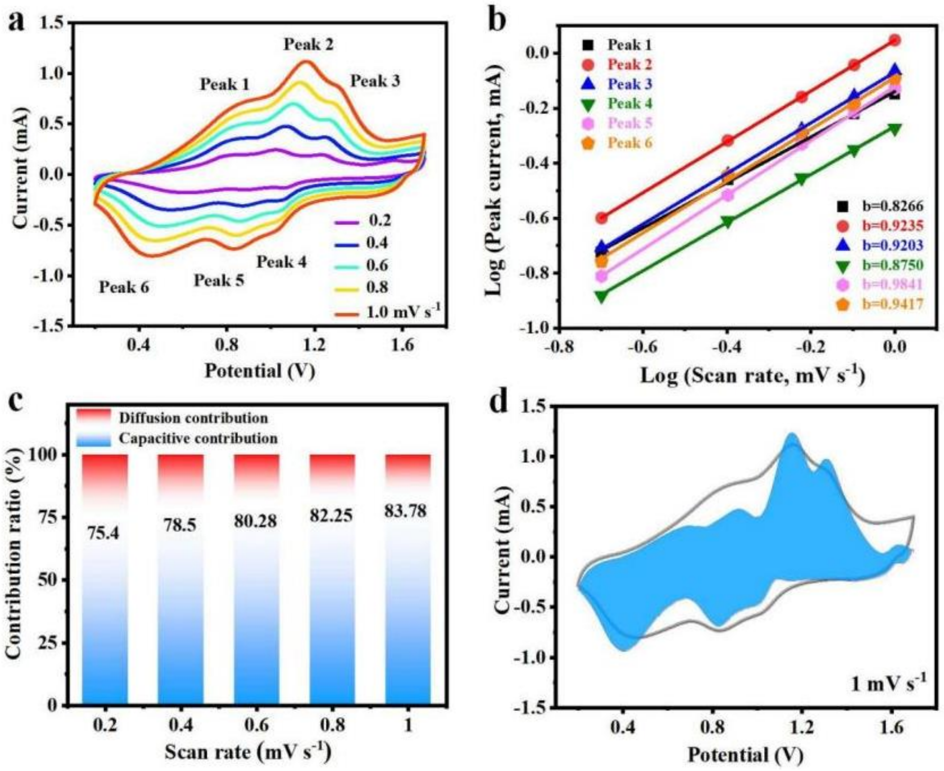

Fig. S20 (**a**) CV curves of V_2O_3/C electrode at scan rates ranging from 0.2 to 1 mV s^{-1} in 50PEG electrolyte. (**b**) The relationship of log (i) versus log (v) curves for peak (1-6) at shown in (**a**). (**c**) Capacitive controlled capacities contributions ratio at various scan rates from 0.2 to 1 mV s^{-1}. (**d**) Cyclic voltammogram showing capacitive controlled (blue region) contribution at 1 mV s^{-1}

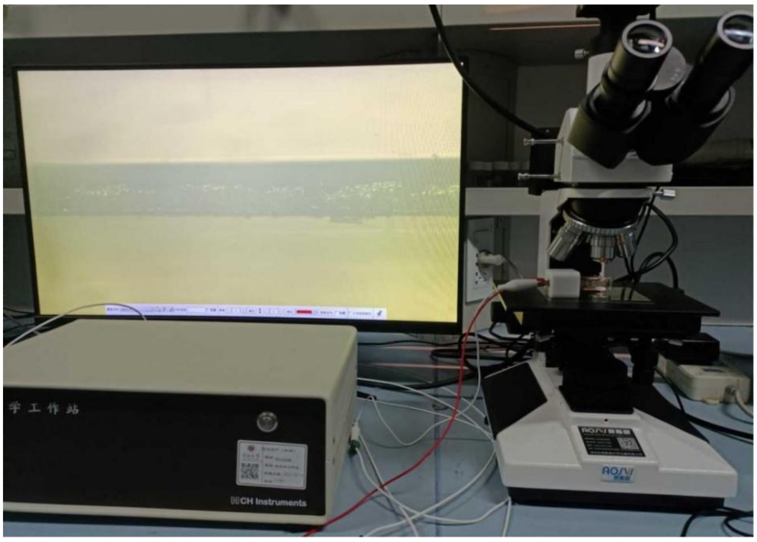

Fig. S21 Digital image of in-situ optical observation device

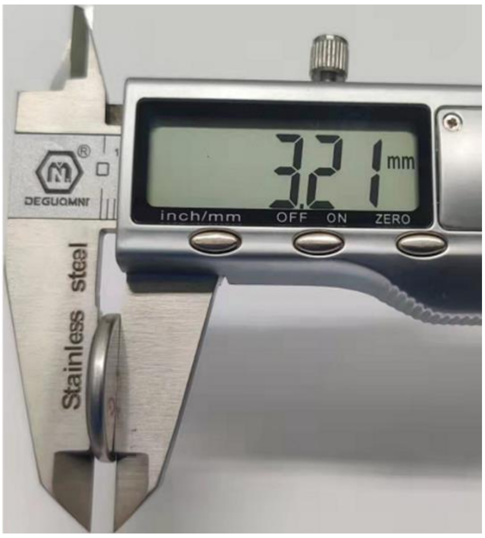

Fig. S22 The thickness of Zn||Zn symmetric cell before cycling

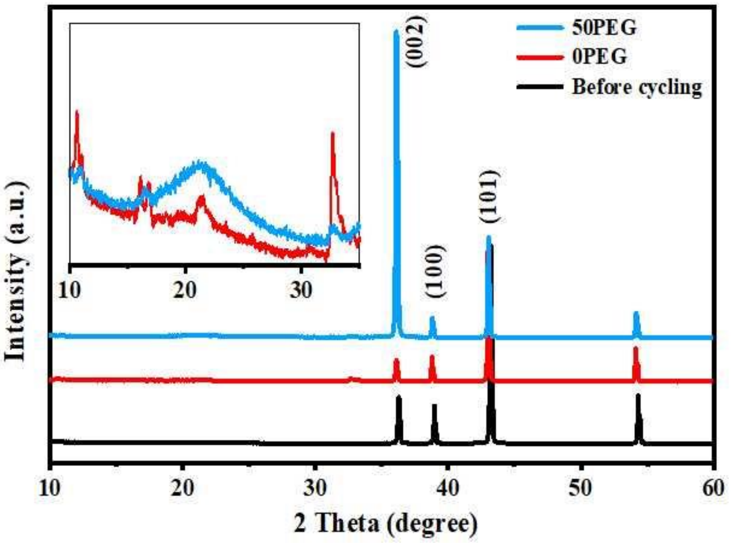

Fig. S23 The comparison of XRD patterns of Zn metal anodes after cycling in 0PEG and 50PEG electrolyte

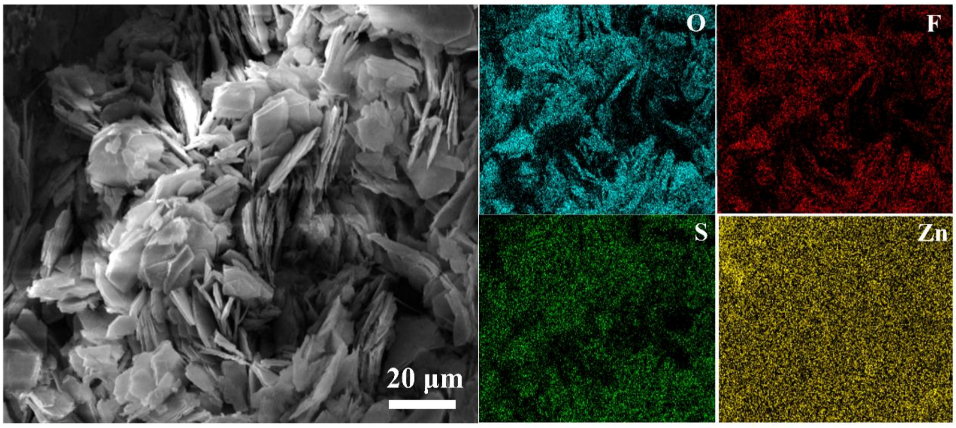

Fig. S24 FESEM image and the corresponding elemental mapping images of Zn metal anode after cycling at the condition of 2 mA cm^{-2} and 2 mAh cm^{-2} for 200 h in the 0PEG electrolyte

Table S1 The comparison of electrochemical performance between our V_2O_3/C cathode with other previously reported cathode materials applied for aqueous Zn-ion batteries

Cathodes	Voltage window	Storage capability	Storage reversibility	Refs.
V_2O_3/C nanosheets	0.2-1.7 V	358.8 mAh g^{-1} at 0.5 A g^{-1}<br>121.8 mAh g^{-1} at 20 A g^{-1}	99% retention after 18000 cycles at 20 A g^{-1}	This work
O_d-MnO_2	1.0-1.8 V	345 mAh g^{-1} at 0.2 A g^{-1}<br>60 mAh g-1 at 30 A g^{-1}	84% retention after 2000 cycles at 5 A g^{-1}	[S1]
MoS_2/graphene	0.2-1.5 V	285.4 mAh g^{-1} at 0.05 A g^{-1}<br>141.6 mAh g-1 at 5 A g^{-1}	88.2% retention after 1800 cycles at 1 A g^{-1}	[S2]
$NaCa_{0.6}V_6O_{16}\cdot 3H_2O$	0.4-1.5 V	347 mAh g^{-1} at 0.1 A g^{-1}<br>154 mAh g^{-1} at 5 A g^{-1}	94% retention after 2000 cycles at 2 A g^{-1}	[S3]
VS_2	0.4-1.0 V	190.3 mAh g^{-1} at 0.05 A g^{-1}<br>115.5 mAh g^{-1} at 2 A g^{-1}	98% retention after 200 cycles at 0.5 A g^{-1}	[S4]
$Zn_3V_3O_8$	0.2-1.6 V	232 mAh g^{-1} at 0.2 A g^{-1}<br>141 mAh g^{-1} at 5 A g^{-1}	72.6% retention after 2000 cycles at 5 A g^{-1}	[S5]
$H_{11}AlV_6O_{23.2}$	0.5-1.7 V	288.4 mAh g^{-1} at 0.1 A g^{-1}<br>163.4 mAh g^{-1} at 5 A g^{-1}	88.6% retention after 7000 cycles at 5 A g^{-1}	[S6]
$Na_3V_2(PO_4)_3$@rGO	0.6-1.8 V	107 mAh g^{-1} at 0.05 A g^{-1}<br>82 mAh g^{-1} at 2 A g^{-1}	75% retention after 200 cycles at 0.5 A g^{-1}	[S7]
$KV_2O_4PO_3\cdot 2H_2O$	0.2-1.8 V	226 mAh g^{-1} at 0.02 A g^{-1}<br>135 mAh g^{-1} at 9 A g^{-1}	75% retention after 3000 cycles at 3 A g^{-1}	[S8]
MoO_{3-x}/MXene	0.25-1.3 V	369.8 mAh g^{-1} at 0.2 A g^{-1}<br>110.6 mAh g^{-1} at 4 A g^{-1}	46.7% retention after 1600 cycles at 4 A g^{-1}	[S9]
MoS_{2-x}	0.25-1.25 V	138.6 mAh g^{-1} at 0.1 A g^{-1}<br>80.6 mAh g^{-1} at 2 A g^{-1}	87.8% retention after 1000 cycles at 1 A g^{-1}	[S10]
$LiV_2(PO_4)_3$	0.2-1.9 V	150 mAh g^{-1} at 0.15 A g^{-1}<br>122 mAh g^{-1} at 9 A g^{-1}	83.3% retention after 4000 cycles at 1.5 A g^{-1}	[S11]
$H_2V_3O_8$/graphene	0.2-1.6 V	336 mAh g^{-1} at 0.1 A g^{-1}<br>215 mAh g^{-1} at 3 A g^{-1}	87% retention after 2000 cycles at 6 A g^{-1}	[S12]
δ-$Ni_{0.25}V_2O_5\cdot nH_2O$	0.3-1.7 V	381 mAh g^{-1} at 0.2 A g^{-1}<br>147 mAh g^{-1} at 5 A g^{-1}	95.7% retention after 1200 cycles at 6 A g^{-1}	[S13]
$VO_2\cdot xH_2O$	0.4-1.4 V	366 mAh g^{-1} at 0.05 A g^{-1}<br>88 mAh g^{-1} at 50 A g^{-1}	89.73% retention after 1000 cycles at 10 A g^{-1}	[S14]
$Li_xV_2O_5\cdot nH_2O$	0.4-1.4 V	470 mAh g^{-1} at 0.5 A g^{-1}<br>170 mAh g^{-1} at 10 A g^{-1}	63% retention after 1000 cycles at 10 A g^{-1}	[S15]
$V_2O_5\cdot nH_2O$/graphene	0.2-1.6 V	372 mAh g^{-1} at 0.3 A g^{-1}<br>248 mAh g^{-1} at 30 A g^{-1}	71% retention after 900 cycles at 6 A g^{-1}	[S16]

Supplementary References

[S1] T. Xiong, Z.G. Yu, H. Wu, Y. Du, Q. Xie et al., Defect engineering of oxygen-deficient manganese oxide to achieve high-performing aqueous zinc ion battery. Adv. Energy Mater. **9**(14), 1803815 (2019). https://doi.org/10.1002/aenm.201803815

[S2] S. Li, Y. Liu, X. Zhao, Q. Shen, W. Zhao et al., Sandwich-like heterostructures of MoS_2 /graphene with enlarged interlayer spacing and enhanced hydrophilicity as high-performance cathodes for aqueous zinc-ion batteries. Adv. Mater. **33**(12), 2007480 (2021). https://doi.org/10.1002/adma.202007480

[S3] K. Zhu, T. Wu, K. Huang, $NaCa_{0.6}V_6O_{16}·3H_2O$ as an ultra-stable cathode for Zn-ion batteries: the roles of pre-inserted dual-cations and structural water in V_3O_8 layer. Adv. Energy Mater. **9**(38), 1901968 (2019). https://doi.org/10.1002/aenm.201901968

[S4] P. He, M. Yan, G. Zhang, R. Sun, L. Chen et al., Layered VS_2 nanosheet-based aqueous Zn ion battery cathode. Adv. Energy Mater. **7**(11), 1601920 (2017). https://doi.org/10.1002/aenm.201601920

[S5] J. Wu, Q. Kuang, K. Zhang, J. Feng, C. Huang et al., Spinel $Zn_3V_3O_8$: a high-capacity zinc supplied cathode for aqueous Zn-ion batteries. Energy Storage Mater. **41**, 297-309 (2021). https://doi.org/10.1016/j.ensm.2021.06.006

[S6] T. Wei, Y. Liu, G. Yang, C. Wang, Aluminum vanadate hollow spheres as zero-strain cathode material for highly reversible and durable aqueous zinc-ion batteries. Energy Storage Mater. **30,** 130-137 (2020). https://doi.org/10.1016/j.ensm.2020.04.039

[S7] P. Hu, T. Zhu, X. Wang, X. Zhou, X. Wei et al., Aqueous $Zn//Zn(CF_3SO_3)_2//Na_3V_2(PO_4)_3$ batteries with simultaneous Zn^{2+}/Na^+ intercalation/de-intercalation. Nano Energy **58**, 492-498 (2019). https://doi.org/10.1016/j.nanoen.2019.01.068

[S8] X. Yang, W. Deng, M. Chen, Y. Wang, C.F. Sun, Mass-producible, quasi-zero-strain, lattice-water-rich inorganic open-frameworks for ultrafast-charging and long-cycling zinc-ion batteries. Adv. Mater. **32**(45), 2003592 (2020). https://doi.org/10.1002/adma.202003592

[S9] J. Shi, Y. Hou, Z. Liu, Y. Zheng, L. Wen et al., The high-performance MoO_{3-x}/MXene cathodes for zinc-ion batteries based on oxygen vacancies and electrolyte engineering. Nano Energy **91**, 106651 (2022). https://doi.org/10.1016/j.nanoen.2021.106651

[S10] W. Xu, C. Sun, K. Zhao, X. Cheng, S. Rawal et al., Defect engineering activating (boosting) zinc storage capacity of MoS_2. Energy Storage Mater. **16**, 527-534 (2019). https://doi.org/10.1016/j.ensm.2018.09.009

[S11] F. Wang, E. Hu, W. Sun, T. Gao, X. Ji et al., A rechargeable aqueous Zn^{2+}-battery with high power density and a long cycle-life. Energy Environ. Sci. **11**(11), 3168-3175 (2018). https://doi.org/10.1039/c8ee01883a

[S12] Q. Pang, C. Sun, Y. Yu, K. Zhao, Z. Zhang et al., $H_2V_3O_8$ nanowire/graphene electrodes for aqueous rechargeable zinc ion batteries with high-rate capability and large capacity. Adv. Energy Mater. **8**(19), 1800144 (2018). https://doi.org/10.1002/aenm.201800144

[S13] J. Li, K. McColl, X. Lu, S. Sathasivam, H. Dong et al., Multi-scale investigations of δ-$Ni_{0.25}V_2O_5·nH_2O$ cathode materials in aqueous zinc-ion batteries. Adv. Energy Mater. **10**(15), 2000058 (2020). https://doi.org/10.1002/aenm.202000058

[S14] N. Liu, X. Wu, L. Fan, S. Gong, Z. Guo et al., Intercalation pseudocapacitive Zn^{2+} storage with hydrated vanadium dioxide toward ultrahigh rate performance. Adv. Mater. **32**(42), 1908420 (2020). https://doi.org/10.1002/adma.201908420

[S15] Y. Yang, Y. Tang, G. Fang, L. Shan, J. Guo et al., Li^{+} intercalated $V_2O_5{\cdot}nH_2O$ with enlarged layer spacing and fast ion diffusion as an aqueous zinc-ion battery cathode. Energy Environ. Sci. **11**(11), 3157-3162 (2018). https://doi.org/10.1039/c8ee01651h

[S16] M. Yan, P. He, Y. Chen, S. Wang, Q. Wei et al., Water-lubricated intercalation in $V_2O_5{\cdot}nH_2O$ for high-capacity and high-rate aqueous rechargeable zinc batteries. Adv. Mater. **30**(1), 1703725 (2018). https://doi.org/10.1002/adma.201703725
